# Biogeography of *Leptospira* in wild animal communities inhabiting the insular ecosystem of the western Indian Ocean islands and neighboring Africa

**DOI:** 10.1038/s41426-018-0059-4

**Published:** 2018-04-04

**Authors:** Muriel Dietrich, Yann Gomard, Erwan Lagadec, Beza Ramasindrazana, Gildas Le Minter, Vanina Guernier, Aude Benlali, Gerard Rocamora, Wanda Markotter, Steven M. Goodman, Koussay Dellagi, Pablo Tortosa

**Affiliations:** 1 Université de La Réunion, UMR PIMIT (Unité Mixte Processus Infectieux en Milieu Insulaire Tropical), INSERM U1187, CNRS UMR 9192, IRD UMR 249, Plateforme CYROI, 2 rue Maxime Rivière, 97490 Sainte Clotilde, La Réunion France; 2 CRVOI - Centre de Recherche et de Veille sur les maladies émergentes dans l’Océan Indien, Sainte Clotilde, 97490 La Réunion France; 3 Department of Medical Virology, Faculty of Health Sciences, Centre for Viral Zoonoses, University of Pretoria, Pretoria, 001 South Africa; 4 Institut Pasteur de Madagascar, 101 Antananarivo, Madagascar; 5 Australian Institute for Tropical Health and Medicine (AITHM), Townsville, 4811 Australia; 6 Island Biodiversity & Conservation Center, University of Seychelles, Anse Royale PO Box 1348, Mahé, Seychelles; 7 Field Museum of Natural History, Chicago, IL 60605 USA; 8 Association Vahatra, 101 Antananarivo, Madagascar; 9 Present Address: Institut Pasteur (Direction Internationale), 75015 Paris, France

## Abstract

Understanding the processes driving parasite assemblages is particularly important in the context of zoonotic infectious diseases. Leptospirosis is a widespread zoonotic bacterial infection caused by pathogenic species of the genus *Leptospira*. Despite a wide range of animal hosts, information is still lacking on the factors shaping *Leptospira* diversity in wild animal communities, especially in regions, such as tropical insular ecosystems, with high host species richness and complex biogeographical patterns. Using a large dataset (34 mammal species) and a multilocus approach at a regional scale, we analyzed the role of both host species diversity and geography in *Leptospira* genetic diversity in terrestrial small mammals (rodents, tenrecs, and shrews) and bats from 10 different islands/countries in the western Indian Ocean (WIO) and neighboring Africa. At least four *Leptospira* spp. (*L*. *interrogans*, *L*. *borgpetersenii*, *L*. *kirschneri*, and *L*. *mayottensis*) and several yet-unidentified genetic clades contributed to a remarkable regional *Leptospira* diversity, which was generally related to the local occurrence of the host species rather than the geography. In addition, the genetic structure patterns varied between *Leptospira* spp., suggesting different evolutionary histories in the region, which might reflect both in situ diversification of native mammals (for *L*. *borgpetersenii*) and the more recent introduction of non-native host species (for *L*. *interrogans*). Our data also suggested that host shifts occurred between bats and rodents, but further investigations are needed to determine how host ecology may influence these events.

## Introduction

Leptospirosis is the most widespread and prevalent zoonosis in the world^1,2^. This emerging disease represents a major health concern on tropical islands, particularly in the Indian Ocean, where some of the highest human incidence rates have been reported^2–4^. The causative organisms are spirochete bacteria of the genus *Leptospira*, which are diverse and divided into 10 currently recognized pathogenic species^5,6^. *Leptospira* infect a wide range of animals that may shed living bacteria in their urine and contribute to environmental contamination and indirectly to human infection. Among wild animals, terrestrial small mammals, especially rodents, are considered to be the main reservoirs for *Leptospira* infections in humans^5^. In addition, evidence of *Leptospira* carriage in different bat species^7–14^ suggests the potential role of these animals in human leptospirosis^15^. However, field-based studies of wild mammal populations often suffer from attempts to culture *Leptospira*, which often fails, hindering further genotyping. Thus, despite its importance for understanding leptospirosis epidemiology, there is currently only limited research on the genetic diversity and evolution of *Leptospira* in terrestrial small mammals and bats^10,16,17^.

The western Indian Ocean (WIO) islands and neighboring countries on the African mainland are characterized by a large diversity of terrestrial small mammals and bats as well as several introduced species^18,19^. In these areas, leptospirosis is endemic and incidences in humans are reported to be among the highest worldwide^3,4,20^. For instance, high incidence rates in humans are reported in the Seychelles^21^ and Mayotte^22^. In contrast, leptospirosis is poorly documented in other regional countries and islands (i.e., Madagascar, Mauritius, Comoros, and South Africa), possibly due to under-diagnosis^20,23,24^.

Epidemiological studies have revealed that depending on the geographic region, humans can be exposed to different *Leptospira* spp. For example, acute human infections on La Réunion are mainly caused by *Leptospirainterrogans*^25^, while on Mayotte, the most prevalent bacterial species identified in acute cases are *L*. *borgpetersenii*, *L*. *kirschneri*, and *L*. *mayottensis*^26^. Several reports have identified an important diversity of *Leptospira* lineages in wild mammals of the region. On Madagascar, introduced rats have been reported to be the only carriers of *L*. *interrogans*^27^, while *L*. *borgpetersenii* and *L*. *kirschneri* infect endemic bats and terrestrial small mammals^10^. On Mayotte, an introduced tenrec species, *Tenrec ecaudatus*, is a main reservoir of *L*. *mayottensis*^28^, which is also found in native tenrec populations in neighboring Madagascar^10^; introduced rats are host to a broader range of pathogenic *Leptospira*^8,28^. On La Réunion, introduced rats are only infected by *L*. *interrogans*^25^, and the partial sequencing of a strain hosted by the endemic bat species *Mormopterus francoismoutoui* revealed a single lineage closely related to *L*. *borgpetersenii*^29^. However, information about *Leptospira* diversity is based on local surveys and often suffers from the use of a single gene for *Leptospira* genetic characterization. As a result of these limitations, the processes shaping the regional *Leptospira* diversity within wild animal communities inhabiting the WIO and neighboring African countries remain unclear.

The aim of the present study is to analyze *Leptospira* genetic diversity and structure within a large range of wild animal hosts at the WIO regional scale and in neighboring Africa. To this end, we used a large range of host species, including terrestrial small mammals and bats. Animals from the WIO region are mostly endemic and those from neighboring Africa are widely distributed species; in addition, we studied the introduced species from both of these regions. We employed an optimized multilocus genotyping approach to specifically examine the respective role of the host and the geography in shaping the regional diversity of this zoonotic bacterium.

## Results

### Samples and genotyping

In total, we used 127 *Leptospira*-positive samples from terrestrial small mammals and bats (Table 1 and Table S1). Our dataset included 34 animal species: 57% were terrestrial small mammals and 43% were bats. Most of the animal species are indigenous to the region, with Nesomyinae and Tenrecidae representing endemic Malagasy adaptive radiations^30,31^. Success in multilocus genotyping was highly variable depending on the samples. A complete multilocus scheme was obtained for only 101 samples (Table 1).

**Table 1:** Details of the *Leptospira*-positive samples from terrestrial small mammals and bats used in the analyses, the number of genetic sequences obtained, and the associated *Leptospira* spp

Order: family	Species	No. of samples	Nb of sequences			*Leptospira* sp
			*rrs2*	*secY*	MLST5	
Madagascar						
Rodentia: Nesomyidae	*Eliurus minor*	15	15	13	13	*Lb*
Afrotheria: Tenrecidae	*Hemicentetes nigriceps*	1	1	1	1	*Lk*
	*Hemicentetes semispinosus*	1	1	1	0	*Lk*
	*Microgale cowani*	2	2	2	1	*Lb, Lm*
	*Microgale dobsoni*	5	5	4	4	*Lm*
	*Microgale longicaudata*	1	1	1	1	*Lb*
	*Microgale majori*	2	2	2	2	*Lb*
	*Microgale principula*	2	2	2	2	*Lb*
Chiroptera: Vespertilionidae	*Myotis goudoti*	1	1	1	1	*Lb*
Chiroptera: Miniopteridae	*Miniopterus gleni*	1	1	1	1	*Lb*
	*Miniopterus griffithsi*	1	1	1	1	*Lb*
	*Miniopterus mahafaliensis*	3	3	3	3	*Lb*
	*Miniopterus majori*	1	1	1	0	*Lb*
	*Miniopterus sororculus*	1	1	1	0	*Lb*
Chiroptera: Molossidae	*Otomops madagascariensis*	1	1	1	1	Lb*
	*Mormopterus jugularis*	2	2	2	1	Lb*
Chiroptera: Pteropodidae	*Pteropus rufus*	1	1	0	0	*L*sp
Chiroptera: Rhinonycteridae	*Triaenops menamena*	5	5	5	3	Li, Lb*, Lsp
Tanzania						
Rodentia: Nesomyidae	*Cricetomys* sp.	7	7	7	7	*Lb, Lk*
Rodentia: Muridae	*Mastomys* sp.	5	5	5	5	*Li, Lb*
Soricomorpha: Soricidae	*Crocidura* sp.	11	11	11	11	*Li, Lb*
Mayotte						
Rodentia: Muridae	*Rattus rattus	8	8	8	8	*Li, Lb*
Afrotheria: Tenrecidae	*Tenrec ecaudatus	8	8	8	8	*Lm*
Seychelles						
Rodentia: Muridae	*Rattus rattus	1	1	1	1	*Li*
Chiroptera: Pteropodidae	*Pteropus seychellensis*	1	1	0	0	*L*sp
La Réunion						
Rodentia: Muridae	*Rattus rattus	3	3	2	2	*Li*
Chiroptera: Molossidae	*Mormopterus francoismoutoui*	4	4	4	4	Lb*
Union of the Comoros						
Chiroptera: Miniopteridae	*Miniopterus griveaudi*	1	1	1	1	*Lb*
Chiroptera: Pteropodidae	*Rousettus obliviosus*	2	2	1	1	*Li, Lb*
Mauritius						
Chiroptera: Molossidae	*Mormopterus acetabulosus*	8	8	8	7	Lb*
South Africa						
Chiroptera: Vespertilionidae	*Glauconycteris variegata*	1	1	0	0	*Lk*
	*Scotophilus dinganii*	1	1	1	1	*Lb*
Chiroptera: Miniopteridae	*Miniopterus natalensis*	8	8	7	6	*Lb, Lk*
Chiroptera: Nycteridae	*Nycteris thebaica*	2	2	2	2	*Lb*
Chiroptera: Pteropodidae	*Rousettus aegyptiacus*	8	8	5	1	Li, Lb*
Swaziland						
Chiroptera: Nycteridae	*Nycteris thebaica*	1	0	1	0	*Li*
Mozambique						
Chiroptera: Miniopteridae	*Miniopterus mossambicus*	1	1	1	1	*Lb*
Total		127	126	115	101	

MLST5 includes the *rrs2*, *secY*, *adk*, *lipL41*, and *icdA* genes. The identification of the different *Leptospira* species is based on single (Figure S2 and S3) and concatenated gene phylogenies (Fig. 3). Introduced species are designated with an asterisk. Two endemic lineages of terrestrial small mammals occur on Madagascar, which include the family Tenrecidae and the subfamily Nesomyinae of the family Nesomyidae. The genus *Cricetomys* belongs to a separate subfamily, the Cricetomyinae, of the Nesomyidae

*Li*=*L. interrogans*, *Lb* = *L*. *borgpetersenii*, Lb* = sequences closely related to *L*. *borgpetersenii*, *Lm* = *L*. *mayottensis*, *Lk* = *L*. *kirschneri*. *L*sp corresponds to unidentified *Leptospira* species.

### Leptospira spp. identification and geographic distribution

**Fig. 1:**
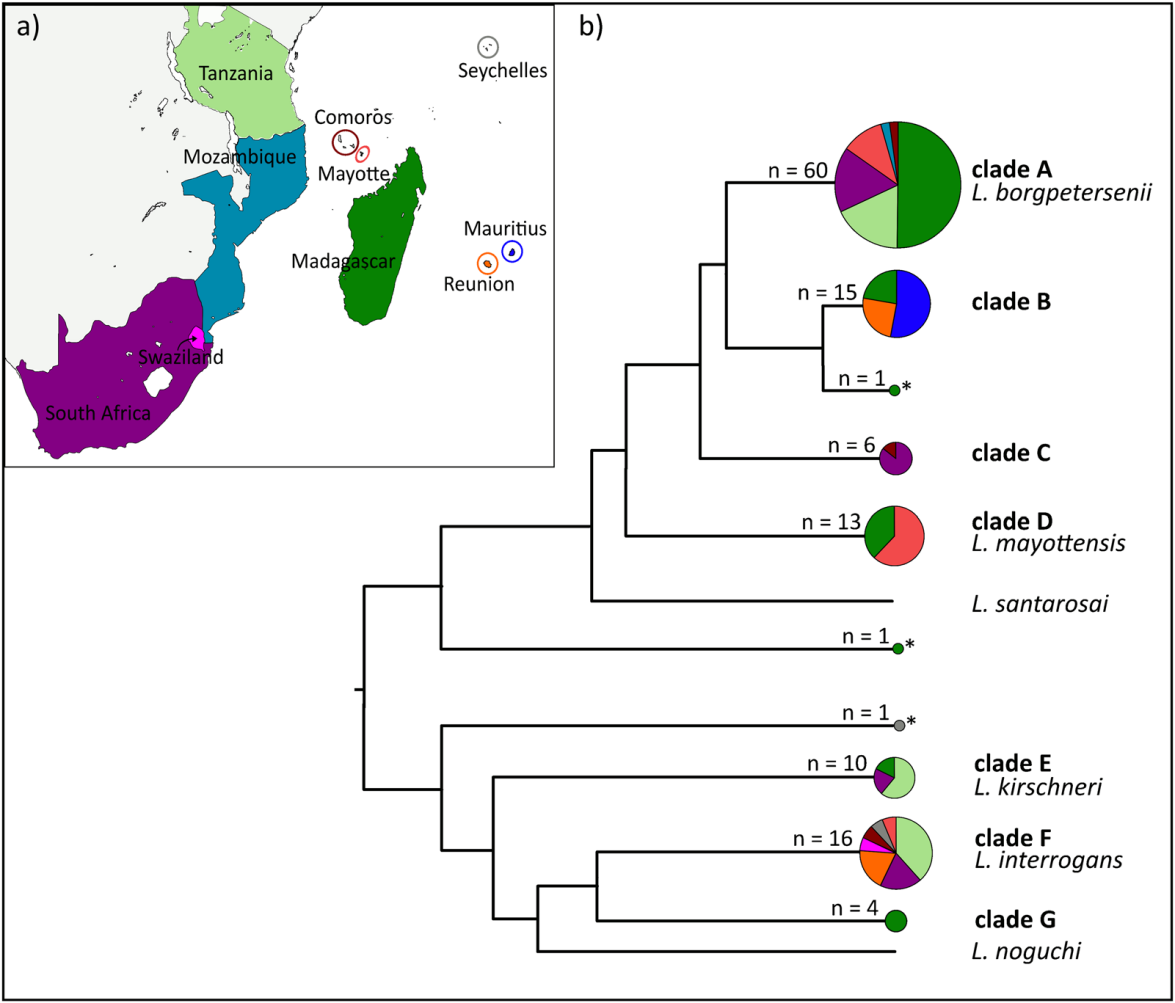
Diversity and geographic distribution of *Leptospira* in terrestrial small mammals and bats of the western Indian Ocean islands and neighboring Africa. **a** Map of the sample locations. **b** Schematic representation of the *Leptospira* phylogenetic relationships based on the maximum-likelihood *secY* and *rrs2* phylogenetic trees (details in Figures S1 and S2). Circle sizes are proportional to the number of samples included for each branch. Colors within the pie charts refer to the different countries as shown on the map. Samples denoted by an asterisk (*) refer to the sequences that could not be assigned to any clade

Among the 127 samples, we used a 473-bp fragment of the *secY* gene for 115 samples to identify *Leptospira* spp. (Figure S2). When *secY* sequences could not be obtained (*n* = 12 samples), we used a 452-bp fragment of the *rrs2* gene instead (Figure S3). The *secY* phylogeny provided a much greater phylogenetic resolution compared to *rrs2*, and some discrepancies between the genes were observed especially for *Leptospira* in the *Rousettus* and *Triaenops* bats (see below). Overall, samples clustered in seven well-supported genetic clades (clades A–G, Fig. 1). Four of these clades grouped with the referenced sequences of *L*. *borgpetersenii* (clade A), *L*. *mayottensis* (clade D), *L*. *kirschneri* (clade E), and *L*. *interrogans* (clade F). The three other clades as well as three single sequences (indicated with an asterisk in Fig. 1) did not cluster with any reference sequences.

Most of the samples grouped in clade A (*L*. *borgpetersenii*), which was widely represented in the region and identified in six of the 10 sampled islands/countries (Fig. 1) from a wide range of animals (i.e., 20 host species, Figure S2). Clade B was closely related to *L*. *borgpetersenii* but was not associated with any reference sequences. This clade was inclusive of Molossidae bats from Madagascar, La Réunion, and Mauritius, except for a *Triaenops* bat from Madagascar. Clade C incorporated *Rousettus* bats from both South Africa and the Comoros. Based on the *secY* phylogeny, it was closely related to *L*. *borgpetersenii*. However, two samples in this clade (samples 1574 and 1577) grouped with *L*. *interrogans* based on the *rrs2* gene (Figure S3).

Clade D, corresponding to *L*. *mayottensis*, included *Leptospira* that infected terrestrial small mammals from Madagascar and Mayotte. Clade E, which was identified as *L*. *kirschneri* based on the reference material, was found in terrestrial small mammals from Madagascar, South Africa, and Tanzania and in *Miniopterus* bats from South Africa. Clade F, assigned to *L*. *interrogans*, was widespread in the region and was identified from seven of the 10 islands/countries. *L*. *interrogans* was found to infect rats (*Rattus rattus*) introduced at all the locations where this species was sampled (i.e., La Réunion, Seychelles, and Mayotte), as well as the insectivorous *Nycteris* bats in Swaziland, and frugivorous *Rousettus* bats in South Africa and the Comoros. Clade G was composed of *Leptospira* that exclusively infected Malagasy bats of the genera *Triaenops* and *Pteropus*; however, the phylogenetic position of *Leptospira* from *Triaenops* varied depending on the gene analyzed. These samples were embedded in the *L*. *interrogans/L*. *noguchi* group depicted in the *secY* phylogeny (Figure S2), although they were more external in the *rrs2* phylogeny. As the *Triaenops* material occupied an intermediate position in both phylogenies, and we did not observe any double peaks in the *Leptospira* sequences for these samples; we considered that the incongruent phylogenies may be the result of the different evolution (e.g., recombination rate) of the studied genes, rather than co-infection, and we maintained these samples in the following multilocus analysis. Finally, a *Pteropus* bat sample from the Seychelles showed an intermediate position in the *rrs2* tree (Figure S3) between *L*. *borgpetersenii* and the *L*. *interrogans*/*L*. *kirschneri/L*. *noguchi* group and did not cluster with any of the reference sequences.

### Genetic diversity and population structure

**Fig. 2:**
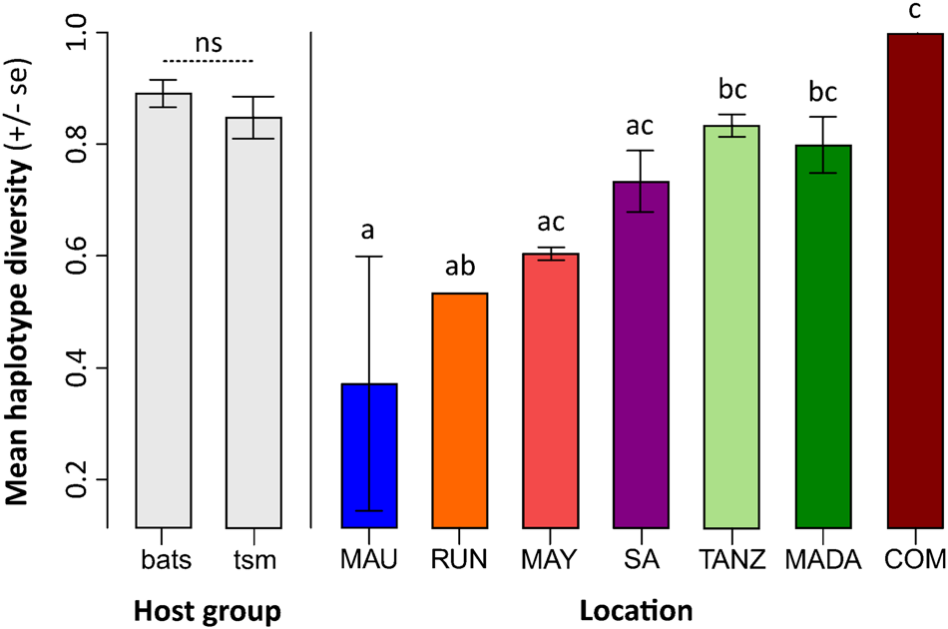
Comparison of *Leptospira* haplotype diversity (± standard error, se) among the host groups and locations in the western Indian Ocean islands and neighboring Africa. “tsm” refers to terrestrial small mammals. “MAU”=Mauritius, “RUN”=La Réunion, “MAY”=Mayotte, “SA” = South Africa, “TANZ” = Tanzania, “MADA” = Madagascar, “COM” = Union of the Comoros. Letters a–c above the bars refer to significantly different averages based upon a Tukey HSD test and “ns” = non-significant. Bars can have more than one letter to reflect the “overlap” between them

We analyzed a complete multilocus scheme of 2252 nucleotides for 101 samples. Haplotype diversity (HD) was calculated, except for the specimens from Mozambique, Swaziland, and the Seychelles, as these locations either contained no sample, or only one sample was available (see Table 1 and Table S5). There was a significant difference in HD among the islands/countries (*p* < 0.001) but not between the terrestrial small mammals and bats (*p* = 0.512, Fig. 2). The highest HD was recorded from the Comoros, but this might be an artifact of the two positive samples from this location being infected by different *Leptospira* spp. High HD was recorded for Madagascar, Tanzania, and South Africa, in contrast with lower HD on La Réunion and Mauritius. However, the Analysis of Molecular Variance (AMOVA) results (Table 2) revealed no significant genetic structure among the islands/countries (*Φ*_CT_ = 0.035, *p* = 0.248) and this was confirmed when the bats and terrestrial small mammals were analyzed separately (Table S4). When examining all the samples, there was not a clear separation between the terrestrial small mammal and bat hosts (*Φ*_CT_ = 0.074, *p* = 0.049), and this was confirmed when analyzing only the Malagasy samples (Table S4). In the AMOVAs, most of the genetic variation was explained by differences among the host genera and species (Table 2 and S4).

**Table 2:** Results of the hierarchical AMOVA analysis

	d.f.	*Φ* Statistics	% Total variation
Geographic structure			
Among islands/countries (South Africa, Madagascar, Comoros, La Réunion, Tanzania and Mayotte)	5	*Φ*_CT_ = 0.035	3.45
Among host species within islands/countries	15	*Φ*_SC_ = 0.600***	57.50
Within populations	71	*Φ*_ST_ = 0.610***	39.05
Structure by host groups (bats vs. terrestrial small mammals)			
Among host groups	1	*Φ*_CT_ = 0.074*	7.42
Among host species within host groups	22	*Φ*_SC_ = 0.613***	56.76
Within populations	77	*Φ*_ST_ = 0.642***	35.83

*p < 0.05

***p < 0.001

**Fig. 3:**
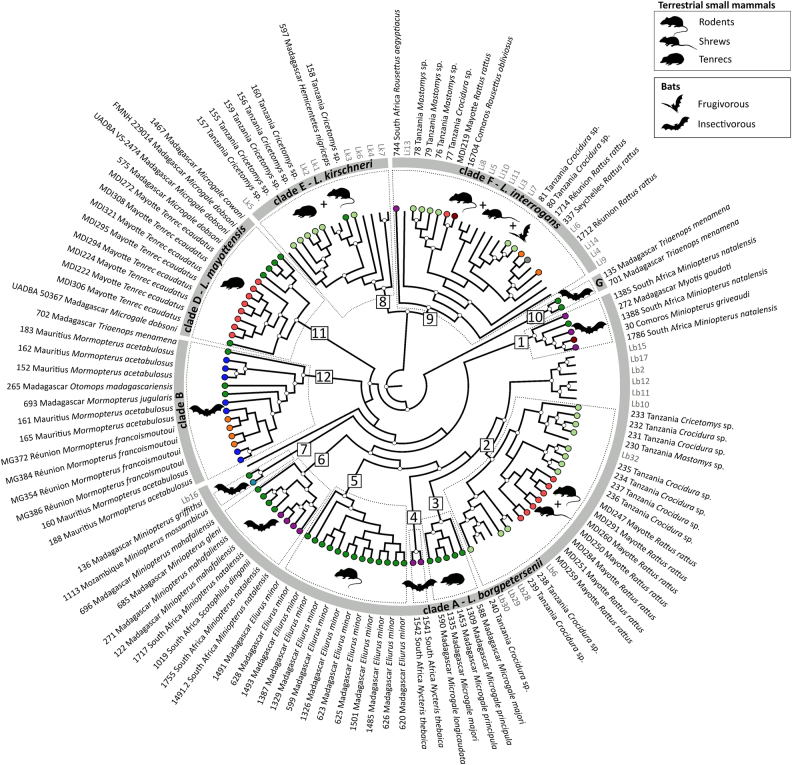
*Leptospira*–host associations in the western Indian Ocean islands and neighboring Africa based on a Bayesian multilocus phylogenetic analysis. The genetic clades identified in Fig. 1 are shown in the gray circle, and the major genetic groups within these clades (squares, numbers 1 to 12) are highlighted by dashed boxes. Animal silhouettes represent host groups. Sequences from the samples specific to this study are in black and coded with the sample ID, geographic location, and host species/genus. Colored circles at the tip of the branches correspond to the geographic locations as shown in Fig. 1. Reference samples are in gray and are coded as follow: Li = *L*. *interrogans*, Lk = *L*. *kirschneri*, and Lb = *L*. *borgpetersenii* (see Table S1 for details). Posterior probabilities higher than 80% are represented by white circles at the nodes. Clade C was not included (refer to Results section for more information)

The coalescent analysis included all the previously identified clades, except clade C (i.e., *Rousettus* samples from South Africa and the Comoros), which was removed because of either failure to obtain the full genotype (samples 6, 1483, 1504, 1564) or suspected co-infections (samples 1577 and 1574, Figure S2 and S3). As shown in Fig. 3, *L*. *borgpetersenii* samples (clade A) were highly diversified and divided into at least seven genetic host-associated clusters (numbers 1–7), with each of these clusters infecting different groups of bats or terrestrial small mammals. Additionally, they were also different from the reference sequences. One exception was those infecting rats introduced on Mayotte and other terrestrial small mammals in Tanzania (see cluster 2), which grouped with strains from Ireland, Indonesia, and Portugal. *L*. *borgpetersenii* infecting rodents and other terrestrial small mammals on Madagascar were separated into two different clusters (numbers 5 and 3, respectively) according to the different host genera (*Eliurus* and *Microgale*). Moreover, *L*. *borgpetersenii* from insectivorous bats (*Miniopterus, Scotophilus*, and *Nycteris*) grouped into four different clusters (numbers 1, 4, 6, and 7), originating from Madagascar, Mozambique, South Africa, and the Comoros. Closely related to *L*. *borgpetersenii*, clade B (number 12) grouped all the Molossidae bat samples from Madagascar (*Otomops madagascariensis*, *Mormopterus jugularis*), La Réunion (*M*. *francoismoutoui*), and Mauritius (*M*. *acetabulosus*) as well as one sample from the Malagasy insectivorous bat *Triaenops menamena* of the family Rhinonycteridae. The two other samples from *T*. *menamena* clustered in a different group (clade G, number 10), which was related, but external, to the other *L*. *borgpetersenii* samples.

In contrast to *L*. *borgpetersenii*, there was less diversity within *L*. *interrogans* and *L*. *kirschneri*, and there was no clear diversification according to the host genus or species, although some sub-structuring was found. For *L*. *interrogans*, terrestrial small mammals and bats were infected with closely related strains. Indeed, this was particularly evident for *L*. *interrogans* infecting rats introduced on Mayotte and *Rousettus* fruit bats in the Comoros (number 9). Finally, *L*. *kirschneri* infecting rodents in Tanzania and tenrecs of the genus *Hemicentetes* on Madagascar were closely related to each other (number 8).

Table 3 shows highly significant *F*_ST_ estimates among the different *Leptospira* clusters. In particular, *Leptospira* from Molossidae bats in clade B and *Triaenops* bats in clade G were highly differentiated from *L*. *borgpetersenii* samples in clade A.

**Table 3:** Pairwise distance (*F*_ST_) among the different genetic *Leptospira* clades in the western Indian Ocean islands and neighboring Africa

		*L. borgpetersenii*-related		*L. mayottensis*	*L. interrogans*	*L. kirschneri*
		Clade B	Clade G	Clade D	Clade E	Clade F
*L. borgpetersenii*	Clade A	0.761***	0.794**	0.845***	0.888***	0.887***
*L. borgpetersenii*-related	Clade B		0.736**	0.853***	0.871***	0.870***
	Clade G			0.917**	0.857**	0.818**
*L. mayottensis*	Clade D				0.971***	0.947***
*L. interrogans*	Clade E					0.858***

Clade C was not included because of the full genotyping failure or suspected co-infections

**p < 0.05

***p  < 0.001

## Discussion

We report here the most comprehensive multilocus sequence analysis study of *Leptospira* in wild animals of the WIO islands and adjacent continental Africa. We reveal a highly diversified assemblage of *Leptospira* in terrestrial small mammals and bats of the region, composed of at least four *Leptospira* species (*L*. *interrogans*, *L*. *borgpetersenii*, *L*. *kirschneri*, *L*. *mayottensis*) and several other *Leptospira* that are apparently not described. Our results highlight that *Leptospira* diversity in wild animals of the WIO is much higher than previously thought and that rodents, tenrecs, shrews, and bats, including introduced and endemic species, contribute to this regional diversity.

### Yet-undescribed diversity

We reported for the first time the presence of *L*. *kirschneri* in *Glauconycteris* and *Miniopterus* bats in South Africa, as well as a closely related *L*. *interrogans* lineage in *Pteropus rufus* on Madagascar. This contrasts with a previous study that did not find antibodies to *Leptospira* in *Pteropus rufus*^32,33^. However, our observations should be taken with caution because they are based on a limited number of samples and only the *rrs2* gene was analyzed. Indeed, Polymerase Chain Reaction (PCR) failures in achieving the complete multilocus (MLST) scheme occurred in 20% of the samples, which limits our capacity to identify these samples to the species level. PCR failures may be related to (i) the small amount of initial material, as we worked with DNA extracts from original samples and not isolates, (ii) potential mismatches in the primer regions, and (ii) the possible absence of the locus coding for surface-expressed proteins (e.g., *lipL41*), as the loss of genes is common in the evolution of pathogenic *Leptospira* strains^34,35^. As the estimation of *Leptospira* diversity is strongly dependent on sample size and the analyzed locus, additional samples are needed for further genetic characterization to confirm that the suggested host species are actual reservoirs of these particular *Leptospira* spp.

Apart from the four already-described *Leptospira* taxa highlighted in our phylogenetic analyses, we also found yet-undescribed genetic clades. For instance, the Bayesian phylogeny and *F*_ST_ values showed that *Leptospira* from Molossidae bats (clade B) and *Triaenops* bats (clade G) were highly genetically divergent from *L*. *borgpetersenii* samples (clade A). Recent investigations on Mayotte led to the description of a new *Leptospira*, *L*. *mayottensis*^6^, which probably originated from Madagascar^10^. In this context, further genetic and serological characterization of Molossidae and *Triaenops*-associated *Leptospira* lineages may lead to the description of new leptospiral species that, so far, are only known from the WIO region.

### Widespread distribution of *L. borgpetersenii* and *L. interrogans*

*L*. *borgpetersenii* and *L*. *interrogans* are largely distributed in our study region. *L*. *interrogans* was found in rodents at all sampled locations, and shrews in Tanzania. This observation is coherent with the fact that *L*. *interrogans* is commonly associated with rodents worldwide^17,27,36,37^. Interestingly, we also reported the presence of *L*. *interrogans*, or closely related lineages, in bats. In particular, *L*. *interrogans* from *Rousettus* in the Union of the Comoros^9^ was almost genetically identical (based on our multilocus scheme) to the one infecting rats in the neighboring island of Mayotte^28^. These results are concordant with a previous study carried out in South America suggesting a rodent–bat transmission and that bats (particularly frugivorous bats) may transmit *L*. *interrogans* to humans^11^. Further investigations are thus required to test whether bats are a natural reservoir of *L*. *interrogans*, and if transmission between terrestrial small mammals and bats may occur. Finally, in our study, *L*. *borgpetersenii* (clade A) was not found in four study regions, namely, Swaziland, Mauritius, La Réunion, and the Seychelles, which might be due to the absence of this strain locally, but more likely is because the typical host species were not sampled in our study. Indeed, on La Réunion, for example, *L*. *borgpetersenii* has been reported in cows and mice^25^, which were hosts that were not analyzed in our study.

### Distinct evolutionary histories?

*Leptospira* diversity in the WIO region was characterized by a lack of spatial structure but high host-associated structure, supporting that *Leptospira* diversity at a given location is strongly correlated to the local host assemblage^10^. Therefore, we can expect that small oceanic islands, that have emerged in situ in recent geological time and had low numbers of native mammal species, would harbor reduced levels of *Leptospira* diversity. This hypothesis is supported by our HD results on La Réunion and Mauritius. As already reported for Madagascar^10,14^, *Leptospira* diversity was regionally shaped by strong host specificity. However, *Leptospira* diversity was not associated with host type (bats vs. terrestrial small mammals), although more samples from these two groups are needed for more definitive conclusions. In contrast, we found that the host genus and species were the main drivers of *Leptospira* diversity with patterns of host specificity varying depending on the *Leptospira* species being considered. Within the *L*. *borgpetersenii* (-like) lineages (clades A and B), there was clear host specificity at the genus and species level. This suggests that strong in situ diversification, favored by the regional island context and rich-endemic mammal fauna of the WIO, is a major driving process in the evolution of the highly diversified *L*. *borgpetersenii*^10,14^. Based on genomic analysis^38^ and environmental studies^16,39^, *L*. *borgpetersenii* is thought to rely more on a direct host-to-host mode of transmission. This may facilitate host adaptation, especially in bat species roosting in close proximity. Co-diversification of *L*. *borgpetersenii* and its hosts is probably a long-term process, as shown by the presence of little or no haplotypic variation in lineages (clade B) on Madagascar, La Réunion, and Mauritius. Indeed, the islands of Mauritius and La Réunion were formed by volcanic activity approximately 5 and 1.8–1.1 million years ago, respectively^40^, and each island has an endemic species of *Mormopterus*, which presumably has had no contact in recent geological time. We hypothesize that the diversification of *Leptospira* sheltered by the three *Mormopterus* likely follows colonization of Mauritius and La Réunion by an ancestral population from Madagascar that arrived with their leptospiral infections. In contrast to these probable endemic *L*. *borgpetersenii* lineages, we found that the only hosts carrying strains found elsewhere (Europe, Indonesia) were rats introduced on Mayotte and different native terrestrial small mammals in Tanzania, suggesting a distinct role of introduced *Rattus* in disseminating cosmopolitan *L*. *borgpetersenii* strains.

In contrast, our results suggest that *L*. *interrogans* (clade F) was less diversified and bat-borne genotypes were genetically related to those of terrestrial small mammals. This suggests a different evolutionary history compared to *L*. *borgpetersenii*. Our hypothesis is that the low diversity of *L*. *interrogans* in the WIO region may be associated with a recent human-associated introduction^41^ of non-native infected rodents (*Rattus* spp. and possibly *Mus* spp.). Additionally, the transmission strategy of *L*. *interrogans*, relying on survival in humid environments^16,38^, may lead to reduced host adaptation and diversification. Additional sampling of bats and terrestrial small mammals in the WIO region and neighboring Africa, with a particular emphasis on the introduced species, will help test our hypothesis of the distinct evolutionary histories between *L*. *borgpetersenii* and *L*. *interrogans*.

### Host ecology and patterns of mono vs. co-infection

We found different patterns of *Leptospira* infection among host species. Both mono-infection (host specificity) and co-infection (multiple clades/species) occurred (Table 1). In bats for instance, the strong host specificity of *Leptospira* in Molossidae bats contrasted with the carriage of multiple *Leptospira* clades/species in the insectivorous *Triaenops menamena*, *Miniopterus* spp., *Nycteris thebaica* or in the frugivorous *Rousettus aegyptiacus*. In this latter species, co-infection with different *Leptospira* spp. was found in certain individuals (Figures S2 and S3). Patterns of co-infection have already been reported from South America in two bat species *Uroderma bilobatum* and *Lonchophylla thomasi*^11^ and from the Comoros in *R*. *obliviosus*^9^. However, the genetic data in other geographical regions are too scarce to ascertain if this is a general pattern in bats. Terrestrial small mammals were also found to be infected with different *Leptospira* species. This was the case for *Rattus* spp. on Mayotte^8^ and three species of terrestrial small mammals from Tanzania^42^. These observations are coherent with several field studies in the rodent populations worldwide^8,16^.

Co-infection may result from the exposure of hosts to diverse environments and should thus be highly dependent of host ecology. Indeed, as highlighted by a recent field study conducted in southeast Asia, different species of *Leptospira* have diverse habitat requirements^16^: *L*. *borgpetersenii* is much more abundant in dry habitats than *L*. *interrogans*, which is restricted to humid habitats, and rodents inhabiting both types of habitats are thus infected by both *Leptospira* species^38^. Humid caves, used as roosting sites by *N*. *thebaica* and *R*. *aegyptiacus* in Swaziland and South Africa, may provide a source of *L*. *interrogans* and may explain why these bat species were found to be infected with both *L*. *borgpetersenii* and *L*. *interrogans*. Further studies should focus on understanding the ecological aspects of the host and the persistence of *Leptospira* species in different environments to disentangle patterns of infection and different epidemiological cycles that may occur in wild animals.

### Human epidemiology

Human leptospirosis on WIO islands and in continental Africa has been generally associated with *Leptospira* spp. circulating in introduced animals^8,27,43^, such as rats or dogs^25^, because these animals are in close proximity to humans and, thus, can act as a source of contaminating *Leptospira*. Our study reveals, however, a wide *Leptospira* diversity circulating in wild animals of the region, with specific lineages associated with the animal taxa present at a given locality. Further investigations should address whether humans may be directly or indirectly (e.g., by a bat-rat transmission cycle) exposed to the local leptospiral diversity sheltered by autochthonous mammals. *Tenrec ecaudatus* has been reported as a main reservoir of *L*. *mayottensis*^28^, which is involved in 15% of human acute cases on Mayotte^26^. A recent study compared *rrs2 Leptospira* sequences from humans on La Réunion to those circulating in the endemic bat species *M*. *francoismoutoui* infected with the *L*. *borgpetersenii* clade B and showed that this bat species was probably not involved in the appearance of clinical cases. However, further studies focusing on public health should investigate the possibility that wild animal-associated strains may lead to symptomless or sub-clinical infections in humans. The positive relationship between host species richness and *Leptospira* diversity presented herein does not necessarily suggest a greater leptospirosis incidence in humans. Indeed, a previous study suggested a negative relationship between mammalian diversity and human incidence of leptospirosis in island ecosystems, which may be explained by both bioregulation and dilution effects^44^. To answer these questions, it will be necessary to improve current techniques for culturing *Leptospira* from field samples and to develop tests for *Leptospira* infection capable of detecting local host-associated *Leptospira* lineages^45^. Such investigations would allow for a greater understanding at the global level of the role of different host/reservoir species in human leptospirosis and the design of improved preventive measures.

## Materials and methods

### Sample collection and dataset

Our study was conducted with *Leptospira* samples from the four host groups (rodents, tenrecs, and shrews—referred to herein as terrestrial small mammals—and bats), from 10 different islands/countries in the WIO and continental Africa (Fig. 1 and Table 1). We used previously published data^9,10,28,42^ (*n* = 75 samples) as well as new samples (*n* = 52, kidney tissue) from La Réunion, Mauritius, Seychelles, South Africa, Swaziland, Mozambique, and Madagascar. For some islands/countries, samples were obtained from a range of localities, and details are provided in Table S1. All samples included in this study were PCR-positive for *Leptospira*, following the protocol described in Dietrich et al.^10^, except for the samples in Tanzania that were positive-cultured strains^42^.

### Ethics statement

New samples were collected in accordance with the terms of the research permits issued by the national authorities; in Madagascar: Ministère des Forêts et de l’Environnement, Madagascar National Parks, Département de Biologie Animale (no. 253/11/MEF/SG/DGF/DCB.SAP/SCB, no. 294/10/MEF/SG/DGF/DCB.SAP/SCB, no. 350/10/MEF/SG/DGF/DCB.SAP/SCB, and no. 68/12/MEF/SG/DGF/DCB.SAP/SCB); in France (La Réunion): Direction de l’Environnement, de l’Aménagement et du Logement; in the Seychelles: Seychelles Bureau of Standards (no. AO157 and AO347) and Ministry of Environment; on Mauritius: National Parks and Conservation Service, in Mozambique: Museum de Historia Naturel and Universidade Eduardo Mondlane; in Swaziland: National Trust Commission for Mlawula Game Reserve; and in South Africa: Limpopo Department of Economic, Environment and Tourism (CPM006806), Gauteng Department of Agriculture, Conservation and Environment, South African National Parks (Kruger National Park–RB/2010/04), North West Department of Economic Development, Environment, Conservation and Tourism (000039 NW-07), Department of Agriculture, Forestry and Fisheries Section 20 permission (12/11/1/1/8). The ethical terms of the research protocol were approved by the CYROI Institutional Animal Care and Use Committee (Comité d’Ethique du CYROI no. 114, IACUC certified by the French Ministry of Higher Education and Research) under accreditation 03387 (LeptOI).

### Leptospira genotyping

The multilocus sequence analysis was performed using a set of five genes according to previous studies investigating *Leptospira* diversity^26,46^. This includes the housekeeping genes *adk*, *secY*, and *icdA*, the 16S rRNA gene *rrs2* and the *lipL41* encoding a surface-expressed protein. Gene sequences were available for some samples from Tanzania, Comoros, Madagascar, and Mayotte^9,10,28,42^. Additional gene sequences for previously published samples as well as genotyping of the new samples (see details in Table S1) were obtained using the amplification conditions described in Dietrich et al.^10^ and the primer list detailed in Table S2. Nucleotide sequences generated in this study were deposited in GenBank under the Accession Numbers KP211557-KP211735, KP211741-KP211743, KP211745-KP211782, KP211785-KP211786, and KT599414-KT599433. Alignments for each gene used in the multilocus analysis are archived at Dryad doi:10.5061/dryad.hj029. Careful manual checking of chromatograms did show double peaks for some samples (see results) suggesting the presence of co-infection within the same individual. Such samples were removed from the multilocus analysis to avoid aberrant concatenation of distinct *Leptospira* lineages infecting the same host.

### Identification of Leptospira spp.

To identify the major *Leptospira* clades and species from the maximum number of samples, we used the *secY* gene, which is highly discriminatory at this taxonomic level^47^ and for which we obtained a large dataset. When *secY* sequences could not be obtained, we used instead the *rrs2* gene to infer *Leptospira* spp. because this gene was most easily amplified. Separate alignments were performed using the CLC Sequence Viewer 6 for the *secY* and *rrs2* genes, including 47 reference sequences representing the major *Leptospira* spp. worldwide (see details in Table S3). JMODELTEST v.2.1.4 was used to search for the best-fit nucleotide substitution model using the Akaike Information Criterion (AIC)^48^, and we constructed phylogenetic trees for the *secY* and *rrs2* genes based on the maximum-likelihood (ML) method with 1000 bootstraps using PHYML^49^.

### Genetic diversity and population structure

To infer the regional genetic structure and evolutionary relationships of *Leptospira*, we used a multilocus analysis, including the five genes described above. Separate alignments were conducted for each gene as mentioned above, and we then employed the coalescent Bayesian inference approach implemented in BEAST v.1.7.3^50^ to infer *Leptospira* diversification. Thirty samples were added to our dataset as reference sequences (Table S3). We used partitioned data with a constant population size coalescent tree prior and unlinked substitution models (a HKY+I+G model of evolution was independently applied to each partition). Runs were initiated on a random starting tree with an unlinked strict clock for each gene (preliminary runs showed that the strict clock better suited the data as the uncorrelated lognormal and exponential relaxed clocks gave ESS values <200 for some parameters and lower posterior means, Figure S1). Analyses were run for 200 × 10^6^ generations, sampling every 1000 generations, with the initial 10% discarded as burn-in. TRACER v.1.5.0^51^ was then used to verify that the effective sample size of each parameter was higher than 200. The sampled posterior trees were summarized using TREEANNOTATOR v.1.6.2 to generate a maximum clade credibility tree (maximum posterior probabilities) and to calculate the mean age and 95% highest posterior density (HPD) interval for each node.

HD was calculated for each gene in DnaSP v5^52^ and the average HD was compared among the locations and host groups (terrestrial small mammals vs. bats) through ANOVA and Tukey HSD (honestly significant difference) post hoc tests. We then used an AMOVA test to determine the significance of an a priori geographic and host-associated structure among samples based on the concatenated sequences using ARLEQUIN v.3.5.1.2^53^. We defined “population” as samples from a single island or country obtained from the same host species (or genus in the case of *Microgale*). In a first analysis, populations were grouped by island/country. In cases when a single sample or a single host was available from a given island/country, these data were excluded (see details in Table 2). The importance of geographic location on *Leptospira* structure was also tested separately for bats and terrestrial small mammals. In a second analysis, populations were grouped by host type (terrestrial small mammals vs. bats). We repeated this second analysis only with the samples from Madagascar, where bats and terrestrial small mammals have been extensively sampled. The *Φ*_CT_, *Φ*_SC_, and Φ_ST_ values were used to estimate the genetic differentiation among the different groups and the significance of the tests was assessed by 1023 permutations of populations between groups.

The level of differentiation among the previously identified *Leptospira* genetic clades/species was estimated by generating *F*_ST_ values in ARLEQUIN v.3.5.1.2 (distance method). The significance of the *F*_ST_ comparisons were assessed using permutation tests (110 permutations per comparison).

## Electronic supplementary material

Supplemental Material(PDF 1432 kb)
