# Supplementary material for: Biogeography of Leptospira in wild animal communities inhabiting the insular ecosystem of the western Indian Ocean islands and neighboring Africa
Source: Emerg Microbes Infect. 2018 Apr 4;7:57. doi: 10.1038/s41426-018-0059-4 (PMC5883017; doi:10.1038/s41426-018-0059-4)
Supplement: Supplementary file 1 — Supplemental Material(PDF 1432 kb) [file 41426_2018_59_MOESM1_ESM.pdf]

Biogeography of *Leptospira* in wild animal communities inhabiting the insular ecosystem of the western Indian Ocean islands and neighboring Africa

Dietrich *et al.* – Supporting Information

Text S1. Animal capture methodology

Animals were captured, manipulated and dispatched in accordance with guidelines accepted by the scientific community and professional mammalogists for the handling of wild mammals [1]. For rodents, tenrecs and shrews, animals were trapped using pitfall buckets and baited-live traps put in place during the night and inspected the following morning. Only baited-live traps were used for sampling on La Réunion and in the Seychelles.

Bats were captured at dusk or during the night using mist nets or harp traps placed in proximity to cave entrances, across presumed flight pathways, or near water sources. On several occasions, a butterfly net was used to obtain bats inside day roost sites.

Captured animals were identified based on different external and cranio-dental characters or using genetic barcoding [2]. Euthanasia of terrestrial small mammals was performed using cervical dislocation, and for bats with the injection of ketamine or inhalation of isoflurane, followed by cardiac puncture. Dissections were performed using sterile utensils. All tissue samples were immediately conserved in liquid nitrogen, and stored at -80°C after transportation to the laboratory.

Table S1. List and details of samples used in this study. A black cross indicates failed PCR.

Sample ID	Museum or field collection number	Island/ Country	Location	Host family or sub-family	Host species	Year	icdA	secY	lipL41	adk	rrs2	Included in the multilocus analysis
6	FMNH 220036	Comoros	Fassi (Grande Comore)	Pteropodidae	*Rousettus obliviosus*	2011	X	X	X	X	This study	NO
30	FMNH 220056	Comoros	Bazimini (Anjouan)	Miniopteridae	*Miniopterus griveaudi*	2011	This study	This study	This study	This study	This study	YES
16704	FMNH 220030	Comoros	Fassi (Grande Comore)	Pteropodidae	*Rousettus obliviosus*	2011	This study	This study	This study	This study	This study	YES
122	FMNH 209278	Madagascar	Toliara	Miniopteridae	*Miniopterus mahafaliensis*	2009	This study	Dietrich *et al*. 2014	Dietrich *et al*. 2014	Dietrich *et al*. 2014	Dietrich *et al*. 2014	YES
135	FMNH 209260	Madagascar	Toliara	Rhinonycteridae	*Triaenops menamena*	2009	This study	This study	This study	This study	This study	YES
136	UADBA 43229	Madagascar	Toliara	Miniopteridae	*Miniopterus griffithsi*	2009	This study	Dietrich *et al*. 2014	Dietrich *et al*. 2014	Dietrich *et al*. 2014	Dietrich *et al*. 2014	YES
265	FMNH 209265	Madagascar	Toliara	Molossidae	*Otomops madagascariensis*	2009	This study	This study	This study	This study	This study	YES
266	FMNH 209258	Madagascar	Toliara	Rhinonycteridae	*Triaenops menamena*	2009	X	This study	X	X	This study	NO
271	FMNH 209276	Madagascar	Toliara	Miniopteridae	*Miniopterus mahafaliensis*	2009	This study	Dietrich *et al*. 2014	Dietrich *et al*. 2014	Dietrich *et al*. 2014	Dietrich *et al*. 2014	YES
272	UADBA SMG-16886	Madagascar	Toliara	Vespertilionidae	*Myotis goudoti*	2009	This study	Dietrich *et al*. 2014	Dietrich *et al*. 2014	Dietrich *et al*. 2014	Dietrich *et al*. 2014	YES
575	UADBA 30869	Madagascar	Ankazomivady	Tenrecidae	*Microgale dobsoni*	2010	This study	Dietrich *et al*. 2014	Dietrich *et al*. 2014	Dietrich *et al*. 2014	Dietrich *et al*. 2014	YES
588	UADBA 30289	Madagascar	Ankazomivady	Tenrecidae	*Microgale majori*	2010	This study	Dietrich *et al*. 2014	Dietrich *et al*. 2014	Dietrich *et al*. 2014	Dietrich *et al*. 2014	YES
590	UADBA 30291	Madagascar	Ankazomivady	Tenrecidae	*Microgale longicaudata*	2010	This study	Dietrich *et al*. 2014	Dietrich *et al*. 2014	Dietrich *et al*. 2014	Dietrich *et al*. 2014	YES
597	UADBA 30850	Madagascar	Ankazomivady	Tenrecidae	*Hemicentetes nigriceps*	2010	This study	Dietrich *et al*. 2014	This study	This study	This study	YES
599	UADBA 30296	Madagascar	Ankazomivady	Nesomyinae	*Eliurus minor*	2010	This study	Dietrich *et al*. 2014	Dietrich *et al*. 2014	Dietrich *et al*. 2014	Dietrich *et al*. 2014	YES
610	UADBA 31054	Madagascar	Ankazomivady	Nesomyinae	*Eliurus minor*	2010	X	X	X	X	This study	NO
620	UADBA 31056	Madagascar	Ankazomivady	Nesomyinae	*Eliurus minor*	2010	This study	Dietrich *et al*. 2014	Dietrich *et al*. 2014	Dietrich *et al*. 2014	Dietrich *et al*. 2014	YES
623	UADBA 31057	Madagascar	Ankazomivady	Nesomyinae	*Eliurus minor*	2010	This study	Dietrich *et al*. 2014	Dietrich *et al*. 2014	Dietrich *et al*. 2014	Dietrich *et al*. 2014	YES
624	UADBA 31058	Madagascar	Ankazomivady	Nesomyinae	*Eliurus minor*	2010	X	X	X	X	This study	NO
625	UADBA 31059	Madagascar	Ankazomivady	Nesomyinae	*Eliurus minor*	2010	This study	Dietrich *et al*. 2014	Dietrich *et al*. 2014	Dietrich *et al*. 2014	Dietrich *et al*. 2014	YES

Sample ID	Museum or field collection number	Island/ Country	Location	Host family or sub-family	Host species	Year	icdA	secY	lipL41	adk	rrs2	Included in the multilocus analysis
626	UADBA 30859	Madagascar	Ankazomivady	Nesomyinae	*Eliurus minor*	2010	This study	Dietrich *et al*. 2014	Dietrich *et al*. 2014	Dietrich *et al*. 2014	Dietrich *et al*. 2014	YES
628	UADBA 30861	Madagascar	Ankazomivady	Nesomyinae	*Eliurus minor*	2010	This study	Dietrich *et al*. 2014	Dietrich *et al*. 2014	Dietrich *et al*. 2014	Dietrich *et al*. 2014	YES
635	UADBA 43253	Madagascar	Fianarantsoa	Miniopteridae	*Miniopterus majori*	2010	X	Dietrich *et al*. 2014	Dietrich *et al*. 2014	Dietrich *et al*. 2014	Dietrich *et al*. 2014	NO
653	UADBA 43265	Madagascar	Fianarantsoa	Miniopteridae	*Miniopterus sororculus*	2010	X	Dietrich *et al*. 2014	Dietrich *et al*. 2014	Dietrich *et al*. 2014	Dietrich *et al*. 2014	NO
685	FMNH 209271	Madagascar	Toliara	Miniopteridae	*Miniopterus gleni*	2009	This study	Dietrich *et al*. 2014	Dietrich *et al*. 2014	Dietrich *et al*. 2014	Dietrich *et al*. 2014	YES
692	UADBA 43211	Madagascar	Toliara	Molossidae	*Mormopterus jugularis*	2009	X	This study	X	X	This study	NO
693	UADBA 43213	Madagascar	Toliara	Molossidae	*Mormopterus jugularis*	2009	This study	This study	This study	This study	This study	YES
696	UADBA 43220	Madagascar	Toliara	Miniopteridae	*Miniopterus mahafaliensis*	2009	This study	Dietrich *et al*. 2014	Dietrich *et al*. 2014	Dietrich *et al*. 2014	Dietrich *et al*. 2014	YES
700	UADBA 43225	Madagascar	Toliara	Rhinonycteridae	*Triaenops menamena*	2009	X	This study	X	X	This study	NO
701	UADBA 43227	Madagascar	Toliara	Rhinonycteridae	*Triaenops menamena*	2009	This study	This study	This study	This study	This study	YES
702	FMNH 209259	Madagascar	Toliara	Rhinonycteridae	*Triaenops menamena*	2009	This study	This study	This study	This study	This study	YES
770	UADBA 31595	Madagascar	Lakato	Tenrecidae	*Hemicentetes semispinosus*	2011	X	Dietrich *et al*. 2014	X	X	This study	NO
1309	UADBA 50057	Madagascar	Ankazomivady	Tenrecidae	*Microgale principula*	2011	This study	Dietrich *et al*. 2014	Dietrich *et al*. 2014	Dietrich *et al*. 2014	Dietrich *et al*. 2014	YES
1310	UADBA 32095	Madagascar	Ankazomivady	Tenrecidae	*Microgale cowani*	2011	X	Dietrich *et al*. 2014	X	X	This study	NO
1326	UADBA 32057	Madagascar	Ankazomivady	Nesomyinae	*Eliurus minor*	2011	This study	Dietrich *et al*. 2014	Dietrich *et al*. 2014	Dietrich *et al*. 2014	Dietrich *et al*. 2014	YES
1329	UADBA 32062	Madagascar	Ankazomivady	Nesomyinae	*Eliurus minor*	2011	This study	Dietrich *et al*. 2014	Dietrich *et al*. 2014	Dietrich *et al*. 2014	Dietrich *et al*. 2014	YES
1335	UADBA 32122	Madagascar	Ankazomivady	Tenrecidae	*Microgale majori*	2011	This study	Dietrich *et al*. 2014	Dietrich *et al*. 2014	Dietrich *et al*. 2014	Dietrich *et al*. 2014	YES
1387	UADBA 50028	Madagascar	Ankazomivady	Nesomyinae	*Eliurus minor*	2011	This study	Dietrich *et al*. 2014	Dietrich *et al*. 2014	Dietrich *et al*. 2014	Dietrich *et al*. 2014	YES
1443	UADBA 32399	Madagascar	Ambovondramanesy	Pteropodidae	*Pteropus rufus*	2012	X	X	X	X	This study	NO
1453	UADBA 32125	Madagascar	Ankazomivady	Tenrecidae	*Microgale principula*	2011	This study	Dietrich *et al*. 2014	Dietrich *et al*. 2014	Dietrich *et al*. 2014	Dietrich *et al*. 2014	YES
1467	UADBA 32101	Madagascar	Ankazomivady	Tenrecidae	*Microgale cowani*	2011	This study	Dietrich *et al*. 2014	Dietrich *et al*. 2014	Dietrich *et al*. 2014	Dietrich *et al*. 2014	YES
1485	UADBA 32069	Madagascar	Ankazomivady	Nesomyinae	*Eliurus minor*	2011	This study	Dietrich *et al*. 2014	Dietrich *et al*. 2014	Dietrich *et al*. 2014	Dietrich *et al*. 2014	YES

Sample ID	Museum or field collection number	Island/ Country	Location	Host family or sub-family	Host species	Year	icdA	secY	lipL41	adk	rrs2	Included in the multilocus analysis
1491	UADBA 50050	Madagascar	Ankazomivady	Nesomyinae	*Eliurus minor*	2011	This study	Dietrich *et al*. 2014	Dietrich *et al*. 2014	Dietrich *et al*. 2014	Dietrich *et al*. 2014	YES
1493	UADBA 32105	Madagascar	Ankazomivady	Nesomyinae	*Eliurus minor*	2011	This study	Dietrich *et al*. 2014	Dietrich *et al*. 2014	Dietrich *et al*. 2014	Dietrich *et al*. 2014	YES
1501	UADBA 32086	Madagascar	Ankazomivady	Nesomyinae	*Eliurus minor*	2011	This study	Dietrich *et al*. 2014	Dietrich *et al*. 2014	Dietrich *et al*. 2014	Dietrich *et al*. 2014	YES
1572	UADBA 50018	Madagascar	Lakato	Tenrecidae	*Microgale dobsoni*	2012	This study	X	X	X	This study	NO
FMNH 229014	FMNH 229014	Madagascar	Ambohitantely	Tenrecidae	*Microgale dobsoni*	2014	This study	Lagadec *et al*. 2016	Lagadec *et al*. 2016	Lagadec *et al*. 2016	Lagadec *et al*. 2016	YES
UADBA 50367	UADBA 50367	Madagascar	Ambohitantely	Tenrecidae	*Microgale dobsoni*	2014	This study	Lagadec *et al*. 2016	Lagadec *et al*. 2016	Lagadec *et al*. 2016	Lagadec *et al*. 2016	YES
UADBA VS-2474	UADBA VS-2474	Madagascar	Ambohitantely	Tenrecidae	*Microgale dobsoni*	2014	This study	Lagadec *et al*. 2016	Lagadec *et al*. 2016	Lagadec *et al*. 2016	Lagadec *et al*. 2016	YES
152	FMNH 213456	Mauritius	Palma	Molossidae	*Mormopterus acetabulosus*	2010	This study	This study	This study	This study	This study	YES
160	FMNH 213464	Mauritius	Palma	Molossidae	*Mormopterus acetabulosus*	2010	This study	This study	This study	This study	This study	YES
161	FMNH 213465	Mauritius	Palma	Molossidae	*Mormopterus acetabulosus*	2010	This study	This study	This study	This study	This study	YES
162	FMNH 213466	Mauritius	Palma	Molossidae	*Mormopterus acetabulosus*	2010	This study	This study	This study	This study	This study	YES
165	FMNH 213469	Mauritius	Palma	Molossidae	*Mormopterus acetabulosus*	2010	This study	This study	This study	This study	This study	YES
183	FMNH 213487	Mauritius	Trois Bras	Molossidae	*Mormopterus acetabulosus*	2010	This study	This study	This study	This study	This study	YES
188	FMNH 213492	Mauritius	Trois Bras	Molossidae	*Mormopterus acetabulosus*	2010	This study	This study	This study	This study	This study	YES
195	FMNH 213499	Mauritius	Camp Thorel	Molossidae	*Mormopterus acetabulosus*	2010	X	This study	X	X	This study	NO
MDI219	MDI 219	Mayotte	Coconi	Muridae	*Rattus rattus*	2014	Lagadec *et al*. 2016	Lagadec *et al*. 2016	Lagadec *et al*. 2016	Lagadec *et al*. 2016	Lagadec *et al*. 2016	YES
MDI222	MDI 222	Mayotte	Coconi	Tenrecidae	*Tenrec ecaudatus*	2014	Lagadec *et al*. 2016	Lagadec *et al*. 2016	Lagadec *et al*. 2016	Lagadec *et al*. 2016	Lagadec *et al*. 2016	YES
MDI224	MDI 224	Mayotte	Coconi	Tenrecidae	*Tenrec ecaudatus*	2014	Lagadec *et al*. 2016	Lagadec *et al*. 2016	Lagadec *et al*. 2016	Lagadec *et al*. 2016	Lagadec *et al*. 2016	YES
MDI247	MDI 247	Mayotte	Kwalé	Muridae	*Rattus rattus*	2014	Lagadec *et al*. 2016	Lagadec *et al*. 2016	Lagadec *et al*. 2016	Lagadec *et al*. 2016	Lagadec *et al*. 2016	YES
MDI250	MDI 250	Mayotte	Kwalé	Muridae	*Rattus rattus*	2014	Lagadec *et al*. 2016	Lagadec *et al*. 2016	Lagadec *et al*. 2016	Lagadec *et al*. 2016	Lagadec *et al*. 2016	YES

Sample ID	Museum or field collection number	Island/ Country	Location	Host family or sub-family	Host species	Year	icdA	secY	lipL41	adk	rrs2	Included in the multilocus analysis
MDI251	MDI 251	Mayotte	Kwalé	Muridae	*Rattus rattus*	2014	Lagadec *et al*. 2016	Lagadec *et al*. 2016	Lagadec *et al*. 2016	Lagadec *et al*. 2016	Lagadec *et al*. 2016	YES
MDI259	MDI 259	Mayotte	Kwalé	Muridae	*Rattus rattus*	2014	Lagadec *et al*. 2016	Lagadec *et al*. 2016	Lagadec *et al*. 2016	Lagadec *et al*. 2016	Lagadec *et al*. 2016	YES
MDI260	MDI 260	Mayotte	Kwalé	Muridae	*Rattus rattus*	2014	Lagadec *et al*. 2016	Lagadec *et al*. 2016	Lagadec *et al*. 2016	Lagadec *et al*. 2016	Lagadec *et al*. 2016	YES
MDI272	MDI 272	Mayotte	Tsoundzou	Tenrecidae	*Tenrec ecaudatus*	2014	Lagadec *et al*. 2016	Lagadec *et al*. 2016	Lagadec *et al*. 2016	Lagadec *et al*. 2016	Lagadec *et al*. 2016	YES
MDI284	MDI 284	Mayotte	Tsoundzou	Muridae	*Rattus rattus*	2014	Lagadec *et al*. 2016	Lagadec *et al*. 2016	Lagadec *et al*. 2016	Lagadec *et al*. 2016	Lagadec *et al*. 2016	YES
MDI291	MDI 291	Mayotte	Tsoundzou	Muridae	*Rattus rattus*	2014	Lagadec *et al*. 2016	Lagadec *et al*. 2016	Lagadec *et al*. 2016	Lagadec *et al*. 2016	Lagadec *et al*. 2016	YES
MDI294	MDI 294	Mayotte	Kwalé	Tenrecidae	*Tenrec ecaudatus*	2014	Lagadec *et al*. 2016	Lagadec *et al*. 2016	Lagadec *et al*. 2016	Lagadec *et al*. 2016	Lagadec *et al*. 2016	YES
MDI295	MDI 295	Mayotte	Kwalé	Tenrecidae	*Tenrec ecaudatus*	2014	Lagadec *et al*. 2016	Lagadec *et al*. 2016	Lagadec *et al*. 2016	Lagadec *et al*. 2016	Lagadec *et al*. 2016	YES
MDI306	MDI 306	Mayotte	Vahibe	Tenrecidae	*Tenrec ecaudatus*	2014	Lagadec *et al*. 2016	Lagadec *et al*. 2016	Lagadec *et al*. 2016	Lagadec *et al*. 2016	Lagadec *et al*. 2016	YES
MDI308	MDI 308	Mayotte	Vahibe	Tenrecidae	*Tenrec ecaudatus*	2014	Lagadec *et al*. 2016	Lagadec *et al*. 2016	Lagadec *et al*. 2016	Lagadec *et al*. 2016	Lagadec *et al*. 2016	YES
MDI321	MDI 321	Mayotte	Vahibe	Tenrecidae	*Tenrec ecaudatus*	2014	Lagadec *et al*. 2016	Lagadec *et al*. 2016	Lagadec *et al*. 2016	Lagadec *et al*. 2016	Lagadec *et al*. 2016	YES
1113	FMNH 213651	Mozambique	Nampula	Miniopteridae	*Miniopterus mossambicus*	2010	This study	This study	This study	This study	This study	YES
494	JCH 001	La Réunion	Sainte Clotilde	Muridae	*Rattus rattus*	2011	X	X	X	X	This study	NO
1712	GLM003	La Réunion	Trois Bassins	Muridae	*Rattus rattus*	2013	This study	This study	This study	This study	This study	YES
1714	GLM005	La Réunion	Trois Bassins	Muridae	*Rattus rattus*	2013	This study	This study	This study	This study	This study	YES
MG354	MG 354	La Réunion	Trois Bassins	Molossidae	*Mormopterus francoismoutoui*	2013	This study	This study	This study	This study	This study	YES
MG372	MG 372	La Réunion	Trois Bassins	Molossidae	*Mormopterus francoismoutoui*	2013	This study	This study	This study	This study	This study	YES
MG384	MG 384	La Réunion	Trois Bassins	Molossidae	*Mormopterus francoismoutoui*	2013	This study	This study	This study	This study	This study	YES
MG386	MG 386	La Réunion	Trois Bassins	Molossidae	*Mormopterus francoismoutoui*	2013	This study	This study	This study	This study	This study	YES
337	GR080	Seychelles	Mahé	Muridae	*Rattus rattus*	2011	This study	This study	This study	This study	This study	YES
YGO42	YGO42	Seychelles	Fairyland	Pteropodidae	*Pteropus seychellensis*	2014	X	X	X	X	This study	NO
744	TM 48522	South Africa	Pafuri, Kruger National Park	Pteropodidae	*Rousettus aegyptiacus*	2010	This study	This study	This study	This study	This study	YES

Sample ID	Museum or field collection number	Island/ Country	Location	Host family or sub-family	Host species	Year	icdA	secY	lipL41	adk	rrs2	Included in the multilocus analysis
846	TM 48630	South Africa	Rocktail Bay, St. Lucia	Vespertilionidae	*Glauconycteris variegata*	2010	X	X	X	X	This study	NO
927	TM 48574	South Africa	Pafuri, Kruger National Park	Pteropodidae	*Rousettus aegyptiacus*	2010	X	X	X	X	This study	NO
1019	TM 48652	South Africa	Kgaswane Nature Reserve, Rustenburg	Vespertilionidae	*Scotophilus dinganii*	2010	This study	This study	This study	This study	This study	YES
1385	UP 1385	South Africa	Irene Caves, Centurion	Miniopteridae	*Miniopterus natalensis*	2011	This study	This study	This study	This study	This study	YES
1388	UP 1388	South Africa	Irene Caves, Centurion	Miniopteridae	*Miniopterus natalensis*	2011	This study	This study	This study	This study	This study	YES
1389	UP 1389	South Africa	Irene Caves, Centurion	Miniopteridae	*Miniopterus natalensis*	2011	X	X	X	X	This study	NO
1471	UP 1471	South Africa	Mahune Cave, Mafefe	Pteropodidae	*Rousettus aegyptiacus*	2012	X	X	X	X	This study	NO
1483	UP 1483	South Africa	Mahune Cave, Mafefe	Pteropodidae	*Rousettus aegyptiacus*	2012	X	This study	X	X	This study	NO
1504	UP 1504	South Africa	Mahune Cave, Mafefe	Pteropodidae	*Rousettus aegyptiacus*	2012	X	This study	X	X	This study	NO
1541	UP 1541	South Africa	Rooiberg, Monate	Nycteridae	*Nycteris thebaica*	2012	This study	This study	This study	This study	This study	YES
1542	UP 1542	South Africa	Rooiberg, Monate	Nycteridae	*Nycteris thebaica*	2012	This study	This study	This study	This study	This study	YES
1564	UP 1564	South Africa	Mahune Cave, Mafefe	Pteropodidae	*Rousettus aegyptiacus*	2012	X	X	X	X	This study	NO
1574	UP 1574	South Africa	Mahune Cave, Mafefe	Pteropodidae	*Rousettus aegyptiacus*	2012	X	This study	X	X	This study	NO
1577	UP 1577	South Africa	Mahune Cave, Mafefe	Pteropodidae	*Rousettus aegyptiacus*	2012	X	This study	X	X	This study	NO
1717	UP 1717	South Africa	Meletse, Thabazimbi	Miniopteridae	*Miniopterus natalensis*	2013	This study	This study	This study	This study	This study	YES
1718	UP 1718	South Africa	Meletse, Thabazimbi	Miniopteridae	*Miniopterus natalensis*	2013	X	This study	X	X	This study	NO
1755	UP 1755	South Africa	Meletse, Thabazimbi	Miniopteridae	*Miniopterus natalensis*	2013	This study	This study	This study	This study	This study	YES
1786	UP 1786	South Africa	Meletse, Thabazimbi	Miniopteridae	*Miniopterus natalensis*	2013	This study	This study	This study	This study	This study	YES
1491.2	UP 1491	South Africa	Mahune Cave, Mafefe	Miniopteridae	*Miniopterus natalensis*	2012	This study	This study	This study	This study	This study	YES
373	UP 373	Swaziland	Mlawula Nature Reserve	Nycteridae	*Nycteris thebaica*	2008	X	This study	X	X	X	NO
76	76	Tanzania	not available	Muridae	*Mastomys* sp.	not available	Nalam *et al.* 2010	Nalam *et al.* 2010	Nalam *et al.* 2010	Nalam *et al.* 2010	Nalam *et al.* 2010	YES

Sample ID	Museum or field collection number	Island/ Country	Location	Host family or sub-family	Host species	Year	icdA	secY	lipL41	adk	rrs2	Included in the multilocus analysis
77	77	Tanzania	not available	Soricidae	*Crocidura* sp.	not available	Nalam *et al*. 2010	Nalam *et al*. 2010	Nalam *et al*. 2010	Nalam *et al*. 2010	Nalam *et al*. 2010	YES
78	78	Tanzania	not available	Muridae	*Mastomys* sp.	not available	Nalam *et al*. 2010	Nalam *et al*. 2010	Nalam *et al*. 2010	Nalam *et al*. 2010	Nalam *et al*. 2010	YES
79	79	Tanzania	not available	Muridae	*Mastomys* sp.	not available	Nalam *et al*. 2010	Nalam *et al*. 2010	Nalam *et al*. 2010	Nalam *et al*. 2010	Nalam *et al*. 2010	YES
80	80	Tanzania	not available	Soricidae	*Crocidura* sp.	not available	Nalam *et al*. 2010	Nalam *et al*. 2010	Nalam *et al*. 2010	Nalam *et al*. 2010	Nalam *et al*. 2010	YES
81	81	Tanzania	not available	Soricidae	*Crocidura* sp.	not available	Nalam *et al*. 2010	Nalam *et al*. 2010	Nalam *et al*. 2010	Nalam *et al*. 2010	Nalam *et al*. 2010	YES
155	155	Tanzania	not available	Cricetomyinae	*Cricetomys* sp.	not available	Nalam *et al*. 2010	Nalam *et al*. 2010	Nalam *et al*. 2010	Nalam *et al*. 2010	Nalam *et al*. 2010	YES
156	156	Tanzania	not available	Cricetomyinae	*Cricetomys* sp.	not available	Nalam *et al*. 2010	Nalam *et al*. 2010	Nalam *et al*. 2010	Nalam *et al*. 2010	Nalam *et al*. 2010	YES
157	157	Tanzania	not available	Cricetomyinae	*Cricetomys* sp.	not available	Nalam *et al*. 2010	Nalam *et al*. 2010	Nalam *et al*. 2010	Nalam *et al*. 2010	Nalam *et al*. 2010	YES
159	159	Tanzania	not available	Cricetomyinae	*Cricetomys* sp.	not available	Nalam *et al*. 2010	Nalam *et al*. 2010	Nalam *et al*. 2010	Nalam *et al*. 2010	Nalam *et al*. 2010	YES
158	158	Tanzania	not available	Cricetomyinae	*Cricetomys* sp.	not available	Nalam *et al*. 2010	Nalam *et al*. 2010	Nalam *et al*. 2010	Nalam *et al*. 2010	Nalam *et al*. 2010	YES
230	230	Tanzania	not available	Muridae	*Mastomys* sp.	not available	Nalam *et al*. 2010	Nalam *et al*. 2010	Nalam *et al*. 2010	Nalam *et al*. 2010	Nalam *et al*. 2010	YES
231	231	Tanzania	not available	Soricidae	*Crocidura* sp.	not available	Nalam *et al*. 2010	Nalam *et al*. 2010	Nalam *et al*. 2010	Nalam *et al*. 2010	Nalam *et al*. 2010	YES
232	232	Tanzania	not available	Soricidae	*Crocidura* sp.	not available	Nalam *et al*. 2010	Nalam *et al*. 2010	Nalam *et al*. 2010	Nalam *et al*. 2010	Nalam *et al*. 2010	YES
233	233	Tanzania	not available	Cricetomyinae	*Cricetomys* sp.	not available	Nalam *et al*. 2010	Nalam *et al*. 2010	Nalam *et al*. 2010	Nalam *et al*. 2010	Nalam *et al*. 2010	YES
234	234	Tanzania	not available	Soricidae	*Crocidura* sp.	not available	Nalam *et al*. 2010	Nalam *et al*. 2010	Nalam *et al*. 2010	Nalam *et al*. 2010	Nalam *et al*. 2010	YES
235	235	Tanzania	not available	Soricidae	*Crocidura* sp.	not available	Nalam *et al*. 2010	Nalam *et al*. 2010	Nalam *et al*. 2010	Nalam *et al*. 2010	Nalam *et al*. 2010	YES
236	236	Tanzania	not available	Soricidae	*Crocidura* sp.	not available	Nalam *et al*. 2010	Nalam *et al*. 2010	Nalam *et al*. 2010	Nalam *et al*. 2010	Nalam *et al*. 2010	YES
237	237	Tanzania	not available	Soricidae	*Crocidura* sp.	not available	Nalam *et al*. 2010	Nalam *et al*. 2010	Nalam *et al*. 2010	Nalam *et al*. 2010	Nalam *et al*. 2010	YES
238	238	Tanzania	not available	Soricidae	*Crocidura* sp.	not available	Nalam *et al*. 2010	Nalam *et al*. 2010	Nalam *et al*. 2010	Nalam *et al*. 2010	Nalam *et al*. 2010	YES
239	239	Tanzania	not available	Muridae	*Mastomys* sp.	not available	Nalam *et al*. 2010	Nalam *et al*. 2010	Nalam *et al*. 2010	Nalam *et al*. 2010	Nalam *et al*. 2010	YES
240	240	Tanzania	not available	Soricidae	*Crocidura* sp.	not available	Nalam *et al*. 2010	Nalam *et al*. 2010	Nalam *et al*. 2010	Nalam *et al*. 2010	Nalam *et al*. 2010	YES

160.2	160	Tanzania	not available	Cricetomyinae	*Cricetomys* sp.	not available	Nalam *et al*. 2010	Nalam *et al*. 2010	Nalam *et al*. 2010	Nalam *et al*. 2010	Nalam *et al*. 2010	YES

Table S2. List of primers and conditions used for the amplification of the five *Leptospira* loci. Primers previously published by Ahmed *et al.* (2006) and Dietrich *et al.* (2014) are marked with an asterisk *[1] and *[2], respectively.

Gene	Primers	Sequence (5' – 3')	T° annealing
rrs2	rrs2F*[1]	CATGCAAGTCAAGCGGAGTA	58°C
	rrs2R*[1]	AGTTGAGCCCGCAGTTTTC	
	rrs2Fd*[2]	CATGCAAGTCRAGCGRAGTA	54°C
	rrs2Rd*[2]	AGTTRAGCYCRCAGTTTTC	
lipL41	lipL41F*[1]	TAGGAAATTGCGCAGCTACA	58°C
	lipL41R*[1]	GCATCGAGAGGAATTAACATCA	
	or lipL41Rd*[2]	GCATCGAGAGGRATYARCATCA	or 54°C
	lipL41F3*[2]	GTCGATGTAGAATATCCGGTATTC	52°C
	lipL41R3*[2]	ACGATTTCTTCGTAACCTTCTTG	
	lipL41F4d*[2]	GATGTAGRATATCCRGTATTC	50°C
	lipL41R5d*[2]	GGAGAAAGTCTRCCYTTGATATA	
icdA	icdAF*[1]	ATCTCCGTTGCACTCTTTGC	58°C
	icdAR*[1]	ACCATCATCATCATCGTCCA	
	icdAF3d	TGGAYTATTATGATTTAGGCGTGGA	
	icdAR2	CTTTTTTGAGATCTCCGGCTTT	54°C
	or icdAR3d	GTGTCYTTTGTMGCRAACCAA	
adk	adkF*[1]	GGGCTGGAAAAGGTACACAA	58°C
	adkR*[1]	ACGCAAGCTCCTTTTGAATC	
	adkFd *[2]	GGGCTGGAAAAGGYACRCAA	56°C
	or adkF3d*[2]	CCTCAGATTTCYACRGGMGATA	or 58°C
	adkR3d*[2]	GCCACTCTTACRCAAGCTCC	
	adkF6d*[2]	GGYTTTCTATYCCTCAGATTTC	50°C
	adkR5d*[2]	CCGACGCCRTTYACTTBAGA	
secY	secYF*[1]	ATGCCGATCATTTTTGCTTC	58°C
	secYR*[1]	CCGTCCCTTAATTTTAGACTTCTTC	
	secYFd*[2]	ATGCCGATCATYTTYGCTTC	52°C
	secYR3*[2]	TTCATGAAGCCTTCATAATTTCTCA	
	secYF4*[2]	GCTTCTTCCTTGATCCTGTTTC	52°C
	secR4d*[2]	TTCATRAAGCCTTCRTAATTTCTCA	

Amplifications were performed with 2 µl of cDNA in a 24 µl reaction mixture containing 12.5 µl of GoTaq Hot Start Green Master Mix 2X (Promega, Madison, WI), 0.5 µl (10 µM) of each primer and 10.5 µl of RNAase free water. The PCR conditions consisted of an initial denaturation step at 95°C for 5 min, followed by 45 cycles of denaturation at 94°C for 30 s, annealing between 50-58°C for 30 s, and extension at 72°C for 1 min, with a final extension at 72°C for 7 min. For the *adk* gene, we performed a second semi-nested PCR (primers adkF3d and adkR3d) for samples exhibiting weak detection signals.

Table S3. Details of the 47 reference strains used in the phylogenetic analyses. All these strains were used in the *secY* (Figure S1) and *rrs2* (Figure S2) phylogenies. The strains

from *L. santarosai* and *L. noguchi* were not included in the multilocus phylogeny (Figure 3), as these *Leptospira* species were not identified in our samples. In addition, four samples (denoted with an asterisk) were not included in the multilocus phylogeny, as the *icdA* gene was not available for these samples.

**Tree ID**	**Label**	**Geographic origin**	**Host species**	**Reference**
Leptospira borgpetersenii				
Lb2	208-Ref8	Italy	Unknown	[3]
Lb6	212-UK22	Indonesia	Unknown	[3]
Lb10	221-QSL91	Kenya	Human	[3]
Lb11	222-QSL104	Bulgaria	Human	[3]
Lb12	223-QSL108	Kenya	Human	[3]
Lb15	226-QSL131	Russia	Human	[3]
Lb16	227-QSL132	Tunisia	Pig	[3]
Lb17	228-QSL134	China	Pig	[3]
Lb28	241-R54	Ireland	Dog	[3]
Lb29	242-R55	Ireland	Pygmy shrew	[3]
Lb30	243-R56	Ireland	Gemsbok	[3]
Lb32	246-R61	Portugal	*Mus musculus*	[3]
Lb38*	st. 200701204	Mayotte	Human	[4]
Leptospira mayottensis				
Lm1*	st. 200901116	Mayotte	Human	[4]
Leptospira interrogans				
Li2*	st. 200901482	Mayotte	Human	[4]
Li3	31-QSL27	Philippines	Rat	[3]
Li4	42-QSL51	Malaysia	Rat	[3]
Li5	44-QSL55	Korea	Mouse	[3]
Li6	62-R7	Brazil	Rat	[3]
Li7	68-R17	Brazil	Cattle	[3]
Li8	69-R17	United Kingdom	Mouse	[3]
Li9	40-QSL47	Peru	Opossum	[3]
Li10	39-QSL46	Malaysia	Human	[3]
Li11	56-QSL105	Sri Lanka	Human	[3]
Li13	22-QSL3	New Guinea	Bandicoot	[3]
Li14	54-QSL95	Australia	Human	[3]
Leptospira santarosai				
Ls1	182-QSL93	Trinidad	Human	[3]
Ls2	184-QSL100	Brazil	Rat	[3]
Ls3	183-QSL99	Peru	Opossum	[3]
Ls4	202-R21	Costa Rica	Human	[3]
Ls5	197-R1	Brazil	Opossum	[3]
Ls6	200-R14	Brazil	Capybara	[3]
Ls7	189-QSL117	USA	Opossum	[3]
Ls8	192-QSL121	Panama	Spiny Rat	[3]
Leptospira kirschneri				
Lk1	146-QSL14	Kenya	Rat	[3]
Lk2	145-QSL12	Russia	Hedgehog	[3]
Lk3	143-QSL9	Bulgaria	Human	[3]
Lk4	152-QSL59	Zimbabwe	Cattle	[3]
Lk5	151-QSL53	Jamaica	Rat	[3]
Lk6	150-QSL44	Ghana	Human	[3]
Lk7	162-R58	Ireland	Mouse	[3]
Lk9*	st. 200801925	Mayotte	Human	[4]
Leptospira noguchi				
Ln1	257-QSL82	Panama	Opossum	[3]
Ln2	254-QSL20	Argentina	Armadillo	[3]

Ln3	255-QSL24	Panama	Rat	[3]
Ln4	256-QSL74	USA	Nutria	[3]
Ln5	253-QSL13	USA	Human	[3]

Table S4. Results of the hierarchical AMOVA analysis. * $p < 0.05$, *** $p < 0.001$.

	d.f.	Φ statistics	% total variation
Geographic structure (bats only)			
Among islands/countries (South Africa, Madagascar, Mauritius, Comoros, La Réunion)	4	Φ_{CT} = 0.067	6.69
Among host species within islands/countries	8	Φ_{SC} = 0.636*	59.38
Within populations	21	Φ_{ST} = 0.661***	33.93
Geographic structure (terrestrial small mammals only)			
Among islands/countries (Madagascar, La Réunion, Tanzania, Mayotte)	3	Φ_{CT} = 0.062	6.22
Among host species within islands/countries	5	Φ_{SC} = 0.580***	54.33
Within populations	56	Φ_{ST} = 0.610***	39.45
Structure by host groups (bats vs. terrestrial small mammals) in Madagascar only			
Among host groups	1	Φ_{CT} = -0.087	8.67
Among host species within host groups	6	Φ_{SC} = 0.577***	62.71
Within populations	27	Φ_{ST} = 0.540***	45.97

Table S5. Haplotype diversity of *Leptospira* in the western Indian Ocean islands and neighboring Africa. Haplotype diversity was not calculated for Mozambique, Swaziland and Seychelles, as either no sample or only one sample was available for these locations. "tsm" refer to terrestrial small mammals.

	**adk**	**icdA**	**lipL41**	**rrs2**	**secY**
**Locations**					
**South Africa**	0.778	0.533	0.756	0.733	0.867
**Madagascar**	0.851	0.794	0.815	0.530	0.845
**Mauritius**	0	0	0.905	0	0.952
**Comoros**	1	1	1	1	1
**La Réunion**	0.533	0.533	0.533	0.533	0.533
**Tanzania**	0.798	0.897	0.877	0.826	0.787
**Host groups**					
**bats**	0.889	0.854	0.938	0.822	0.953
**tsm**	0.871	0.863	0.882	0.693	0.880

Figure S1. Mean posterior values obtained with BEAST for the strict clock, the uncorrelated relaxed lognormal and exponential clocks.

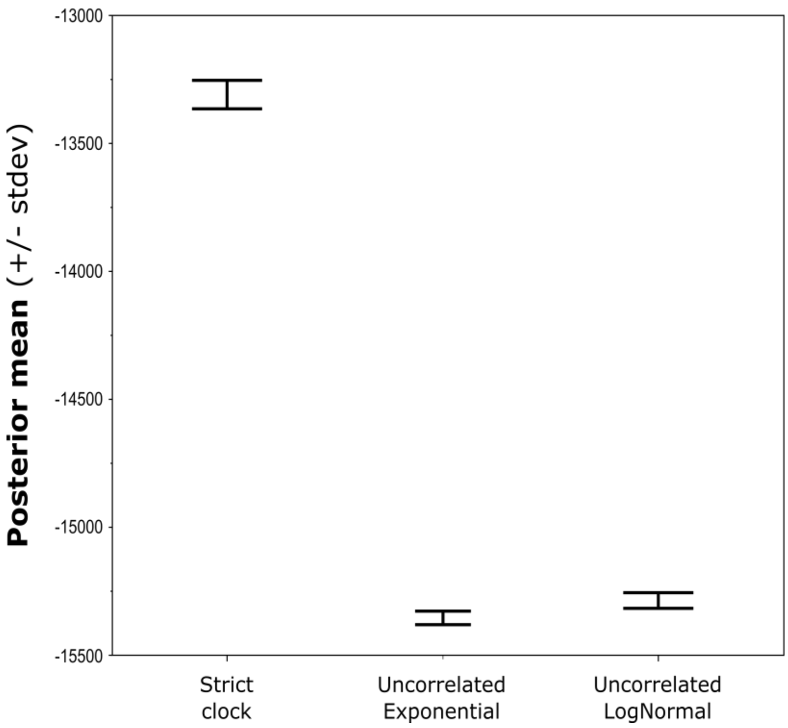

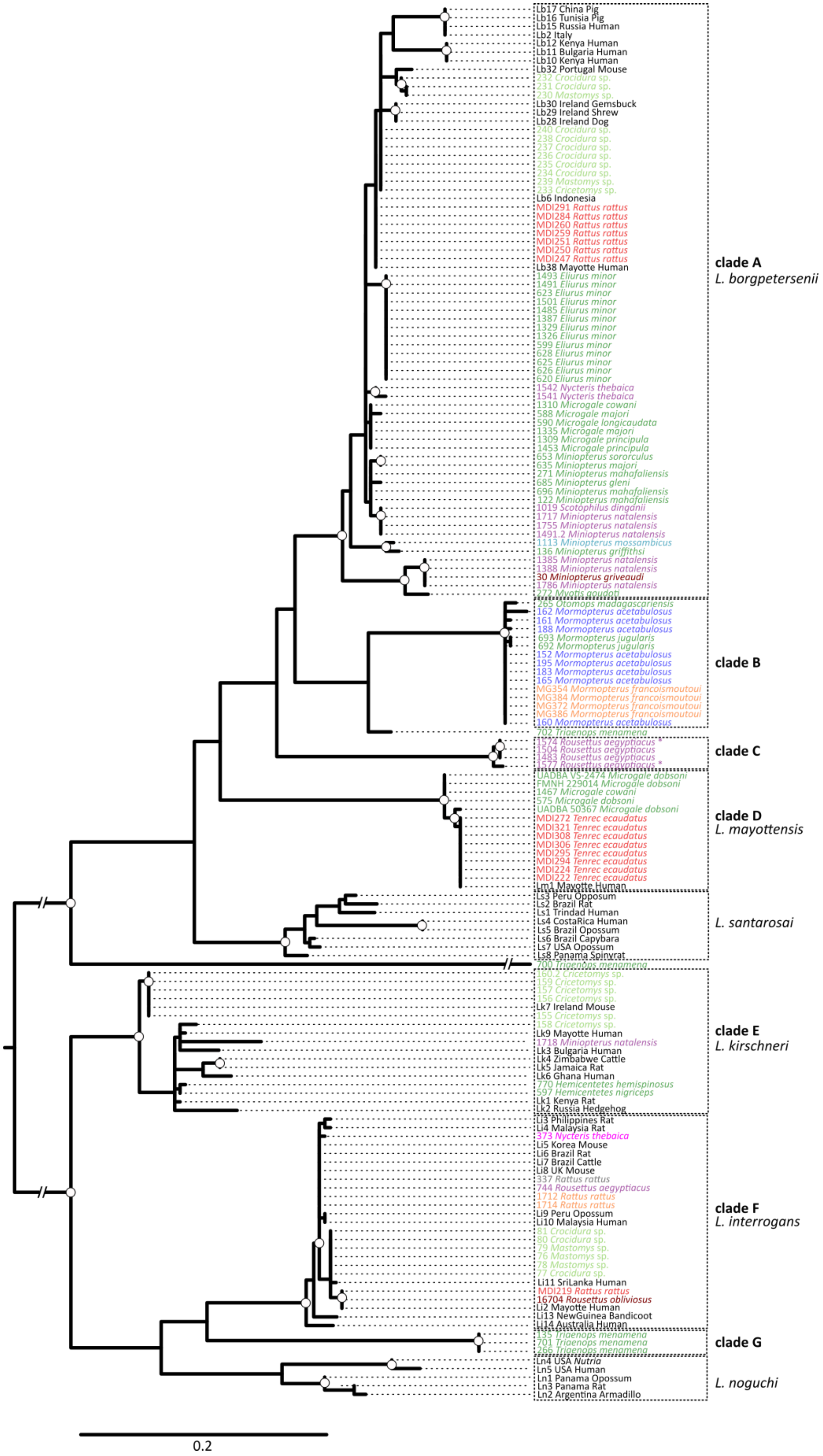

Lb17 China Pig
Lb16 Tunisia Pig
Lb15 Russia Human
Lb2 Italy
Lb12 Kenya Human
Lb11 Bulgaria Human
Lb10 Kenya Human
Lb32 Portugal Mouse
232 Crocidura sp.
231 Crocidura sp.
230 Mastomys sp.
Lb30 Ireland Gemsbuck
Lb29 Ireland Shrew
Lb28 Ireland Dog
240 Crocidura sp.
238 Crocidura sp.
237 Crocidura sp.
236 Crocidura sp.
235 Crocidura sp.
234 Crocidura sp.
239 Mastomys sp.
233 Cricetomys sp.
Lb6 Indonesia
MDI291 Rattus rattus
MDI284 Rattus rattus
MDI260 Rattus rattus
MDI259 Rattus rattus
MDI251 Rattus rattus
MDI250 Rattus rattus
MDI247 Rattus rattus
Lb38 Mayotte Human
1493 Eliurus minor
1491 Eliurus minor
623 Eliurus minor
1501 Eliurus minor
1485 Eliurus minor
1387 Eliurus minor
1329 Eliurus minor
1326 Eliurus minor
599 Eliurus minor
628 Eliurus minor
625 Eliurus minor
626 Eliurus minor
620 Eliurus minor
1542 Nycteris thebaica
1541 Nycteris thebaica
1310 Microgale cowani
588 Microgale majori
590 Microgale longicaudata
1335 Microgale majori
1309 Microgale principula
1453 Microgale principula
653 Miniopterus sororculus
635 Miniopterus majori
271 Miniopterus mahafaliensis
685 Miniopterus gleni
696 Miniopterus mahafaliensis
122 Miniopterus mahafaliensis
1019 Scotophilus dinganii
1717 Miniopterus natalensis
1755 Miniopterus natalensis
1491.2 Miniopterus natalensis
1113 Miniopterus mossambicus
136 Miniopterus griffithsi
1385 Miniopterus natalensis
1388 Miniopterus natalensis
30 Miniopterus griveaudi
1786 Miniopterus natalensis
272 Myotis goudoti
clade A
L. borgpetersenii
265 Otomops madagascariensis
162 Mormopterus acetabulosus
161 Mormopterus acetabulosus
188 Mormopterus acetabulosus
693 Mormopterus jugularis
692 Mormopterus jugularis
152 Mormopterus acetabulosus
195 Mormopterus acetabulosus
183 Mormopterus acetabulosus
165 Mormopterus acetabulosus
MG354 Mormopterus francoismoutoui
MG384 Mormopterus francoismoutoui
MG372 Mormopterus francoismoutoui
MG386 Mormopterus francoismoutoui
160 Mormopterus acetabulosus
clade B
702 Triaenops menamena
1574 Rousettus aegyptiacus *
1504 Rousettus aegyptiacus
1483 Rousettus aegyptiacus
1577 Rousettus aegyptiacus *
clade C
UADBA VS-2474 Microgale dobsoni
FMNH 229014 Microgale dobsoni
1467 Microgale cowani
575 Microgale dobsoni
UADBA 50367 Microgale dobsoni
MDI272 Tenrec ecaudatus
MDI321 Tenrec ecaudatus
MDI308 Tenrec ecaudatus
MDI306 Tenrec ecaudatus
MDI295 Tenrec ecaudatus
MDI294 Tenrec ecaudatus
MDI224 Tenrec ecaudatus
MDI222 Tenrec ecaudatus
Lm1 Mayotte Human
clade D
L. mayottensis
Ls3 Peru Opposum
Ls2 Brazil Rat
Ls1 Trindad Human
Ls4 CostaRica Human
Ls5 Brazil Opossum
Ls6 Brazil Capybara
Ls7 USA Opossum
Ls8 Panama Spinyrat
L. santarosai
700 Triaenops menamena
160.2 Cricetomys sp.
159 Cricetomys sp.
157 Cricetomys sp.
156 Cricetomys sp.
Lk7 Ireland Mouse
155 Cricetomys sp.
158 Cricetomys sp.
Lk9 Mayotte Human
1718 Miniopterus natalensis
Lk3 Bulgaria Human
Lk4 Zimbabwe Cattle
Lk5 Jamaica Rat
Lk6 Ghana Human
770 Hemicentetes hemispinosus
597 Hemicentetes nigriceps
Lk1 Kenya Rat
Lk2 Russia Hedgehog
clade E
L. kirschneri
Li3 Philippines Rat
Li4 Malaysia Rat
373 Nycteris thebaica
Li5 Korea Mouse
Li6 Brazil Rat
Li7 Brazil Cattle
Li8 UK Mouse
337 Rattus rattus
744 Rousettus aegyptiacus
1712 Rattus rattus
1714 Rattus rattus
Li9 Peru Opossum
Li10 Malaysia Human
81 Crocidura sp.
80 Crocidura sp.
79 Mastomys sp.
76 Mastomys sp.
78 Mastomys sp.
77 Crocidura sp.
Li11 SriLanka Human
MDI219 Rattus rattus
16704 Rousettus obliviosus
Li2 Mayotte Human
Li13 NewGuinea Bandicoot
Li14 Australia Human
clade F
L. interrogans
135 Triaenops menamena
701 Triaenops menamena
266 Triaenops menamena
clade G
Ln4 USA Nutria
Ln5 USA Human
Ln1 Panama Opossum
Ln3 Panama Rat
Ln2 Argentina Armadillo
L. noguchi
0.2

Figure S2. Maximum likelihood phylogenetic tree based on the analysis of *secY* gene fragment. Taxa are colored according to the location (see Figure 1). Taxa with an asterisk (*) were suspected of co-infection. Reference sequences are indicated in black. Bootstrap values higher than 75% are represented by white circles. The boxes with dashed lines correspond to the major genetic clades. The tree was constructed with a TIM3+I+G model.

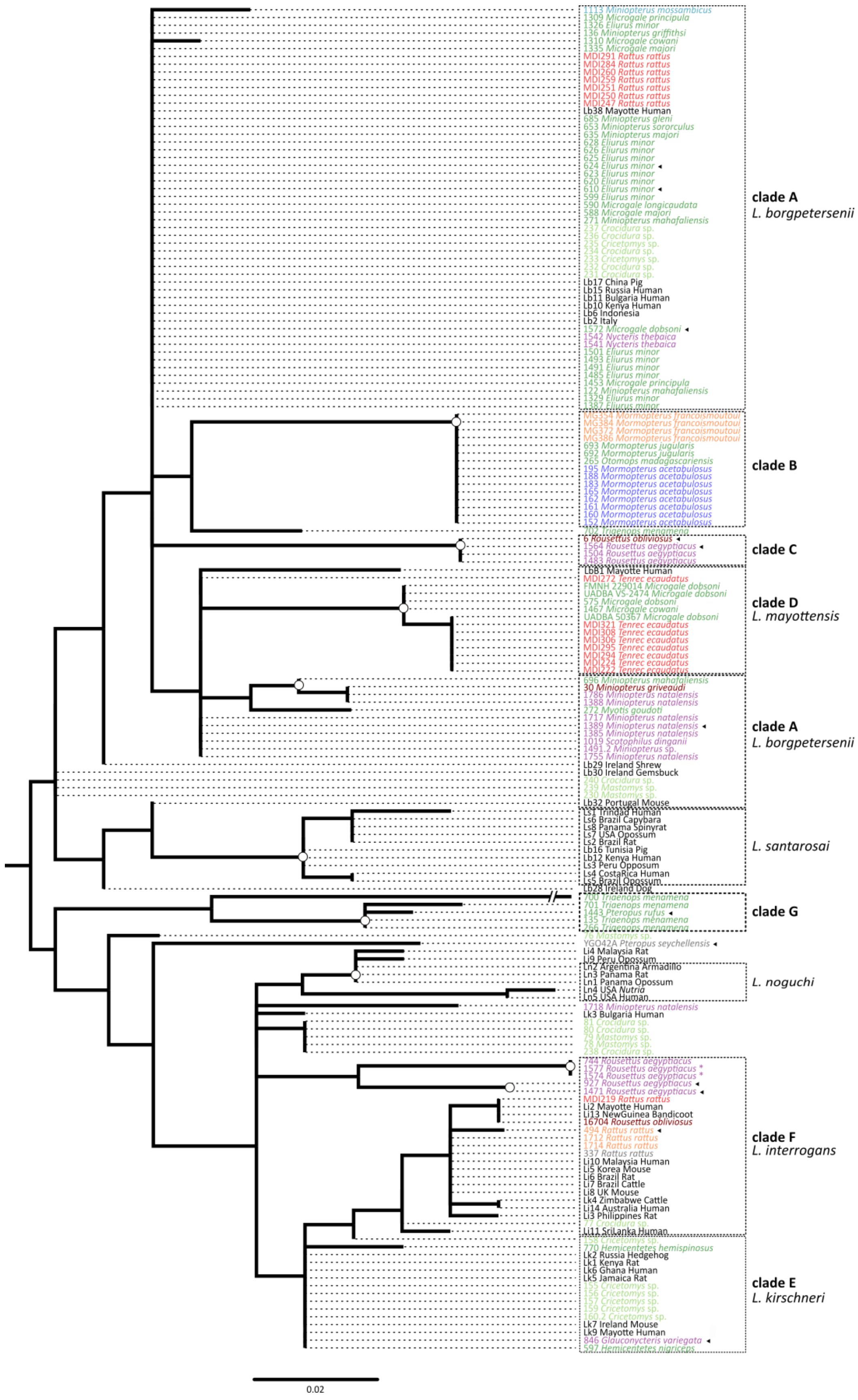

1113 Miniopterus mossambicus
1309 Microgale principula
1326 Eliurus minor
136 Miniopterus griffithsi
1310 Microgale cowani
1335 Microgale majori
MDI291 Rattus rattus
MDI284 Rattus rattus
MDI260 Rattus rattus
MDI259 Rattus rattus
MDI251 Rattus rattus
MDI250 Rattus rattus
MDI247 Rattus rattus
Lb38 Mayotte Human
685 Miniopterus gleni
653 Miniopterus sororculus
635 Miniopterus majori
628 Eliurus minor
626 Eliurus minor
625 Eliurus minor
624 Eliurus minor
623 Eliurus minor
620 Eliurus minor
610 Eliurus minor
599 Eliurus minor
590 Microgale longicaudata
588 Microgale majori
271 Miniopterus mahafaliensis
237 Crocidura sp.
236 Crocidura sp.
235 Cricetomys sp.
234 Crocidura sp.
233 Cricetomys sp.
232 Crocidura sp.
231 Crocidura sp.
Lb17 China Pig
Lb15 Russia Human
Lb11 Bulgaria Human
Lb10 Kenya Human
Lb6 Indonesia
Lb2 Italy
1572 Microgale dobsoni
1542 Nycteris thebaica
1541 Nycteris thebaica
1501 Eliurus minor
1493 Eliurus minor
1491 Eliurus minor
1485 Eliurus minor
1453 Microgale principula
122 Miniopterus mahafaliensis
1329 Eliurus minor
1387 Eliurus minor
clade A
L. borgpetersenii
MG354 Mormopterus francoismoutoui
MG384 Mormopterus francoismoutoui
MG372 Mormopterus francoismoutoui
MG386 Mormopterus francoismoutoui
693 Mormopterus jugularis
692 Mormopterus jugularis
265 Otomops madagascariensis
195 Mormopterus acetabulosus
188 Mormopterus acetabulosus
183 Mormopterus acetabulosus
165 Mormopterus acetabulosus
162 Mormopterus acetabulosus
161 Mormopterus acetabulosus
160 Mormopterus acetabulosus
152 Mormopterus acetabulosus
clade B
702 Triaenops menamena
6 Rousettus obliviosus
1564 Rousettus aegyptiacus
1504 Rousettus aegyptiacus
1483 Rousettus aegyptiacus
clade C
LbB1 Mayotte Human
MDI272 Tenrec ecaudatus
FMNH 229014 Microgale dobsoni
UADBA VS-2474 Microgale dobsoni
575 Microgale dobsoni
1467 Microgale cowani
UADBA 50367 Microgale dobsoni
MDI321 Tenrec ecaudatus
MDI308 Tenrec ecaudatus
MDI306 Tenrec ecaudatus
MDI295 Tenrec ecaudatus
MDI294 Tenrec ecaudatus
MDI224 Tenrec ecaudatus
MDI222 Tenrec ecaudatus
clade D
L. mayottensis
696 Miniopterus mahafaliensis
30 Miniopterus griveaudi
1786 Miniopterus natalensis
1388 Miniopterus natalensis
272 Myotis goudoti
1717 Miniopterus natalensis
1389 Miniopterus natalensis
1385 Miniopterus natalensis
1019 Scotophilus dinganii
1491.2 Miniopterus sp.
1755 Miniopterus natalensis
Lb29 Ireland Shrew
Lb30 Ireland Gemsbuck
240 Crocidura sp.
239 Mastomys sp.
230 Mastomys sp.
Lb32 Portugal Mouse
clade A
L. borgpetersenii
Ls1 Trinidad Human
Ls6 Brazil Capybara
Ls8 Panama Spinyrat
Ls7 USA Opossum
Ls2 Brazil Rat
Lb16 Tunisia Pig
Lb12 Kenya Human
Ls3 Peru Opposum
Ls4 CostaRica Human
Ls5 Brazil Opossum
Lb28 Ireland Dog
L. santarosai
700 Triaenops menamena
701 Triaenops menamena
1443 Pteropus rufus
135 Triaenops menamena
266 Triaenops menamena
clade G
76 Mastomys sp.
YGO42A Pteropus seychellensis
Li4 Malaysia Rat
Li9 Peru Opossum
Ln2 Argentina Armadillo
Ln3 Panama Rat
Ln1 Panama Opossum
Ln4 USA Nutria
Ln5 USA Human
L. noguchi
1718 Miniopterus natalensis
Lk3 Bulgaria Human
81 Crocidura sp.
80 Crocidura sp.
79 Mastomys sp.
78 Mastomys sp.
238 Crocidura sp.
744 Rousettus aegyptiacus
1577 Rousettus aegyptiacus *
1574 Rousettus aegyptiacus *
927 Rousettus aegyptiacus
1471 Rousettus aegyptiacus
MDI219 Rattus rattus
Li2 Mayotte Human
Li13 NewGuinea Bandicoot
16704 Rousettus obliviosus
494 Rattus rattus
1712 Rattus rattus
1714 Rattus rattus
337 Rattus rattus
Li10 Malaysia Human
Li5 Korea Mouse
Li6 Brazil Rat
Li7 Brazil Cattle
Li8 UK Mouse
Lk4 Zimbabwe Cattle
Li14 Australia Human
Li3 Philippines Rat
77 Crocidura sp.
Li11 SriLanka Human
clade F
L. interrogans
158 Cricetomys sp.
770 Hemicentetes hemispinosus
Lk2 Russia Hedgehog
Lk1 Kenya Rat
Lk6 Ghana Human
Lk5 Jamaica Rat
155 Cricetomys sp.
156 Cricetomys sp.
157 Cricetomys sp.
159 Cricetomys sp.
160.2 Cricetomys sp.
Lk7 Ireland Mouse
Lk9 Mayotte Human
846 Glauconycteris variegata
597 Hemicentetes nigriceps
clade E
L. kirschneri
0.02

Figure S3. Maximum likelihood phylogenetic tree based on the analysis of *rrs2* gene fragment. Taxa are colored according to the location (see Figure 1). The 12 sequences added to the *rrs2* phylogeny, compared to the *secY* phylogeny, are denoted by a black triangle (◂). Taxa with an asterisk (*) were suspected of co-infection. Reference sequences are indicated in black. Clades identified with the *secY* phylogeny (figure S1) are indicated on the tree. Bootstrap values higher than 75% are represented by white circles. The tree was constructed with a TIM3+I+G model.

References

1 Sikes RS, The Animal Care and use C of the AS of M. 2016 Guidelines of the American Society of Mammalogists for the use of wild mammals in research and education. *J Mammal* 2016; **97**: 663–688.

2 Folmer O, Black M, Hoeh W, Lutz R, Vrijenhoek R. DNA primers for amplification of mitochondrial cytochrome c oxidase subunit I from diverse metazoan invertebrates. *Mol Mar Biol Biotechnol* 1994; **3**: 294–299.

3 Ahmed N, Devi SM, Valverde MDLÁ, Vijayachari P, Machang RS, Ellis WA *et al*. Multilocus sequence typing method for identification and genotypic classification of pathogenic *Leptospira* species. *Ann Clin Microbiol Antimicrob* 2006; **5**: 28.

4 Bourhy P, Collet L, Lernout T, Zinini F, Hartskeerl RA, van der Linden H *et al*. Human *Leptospira* isolates circulating in Mayotte (Indian Ocean) have unique serological and molecular features. *J Cinical Microbiol* 2012; **50**: 307–11.
